# Supplementary material for: Impact of adverse events during community-wide mass drug administration for soil-transmitted helminths on subsequent participation–a Theory of Planned Behaviour analysis
Source: PLoS Negl Trop Dis. 2023 Mar 14;17(3):e0011148. doi: 10.1371/journal.pntd.0011148 (PMC10013912; doi:10.1371/journal.pntd.0011148)
Supplement: S2 File — (DOCX) [file pntd.0011148.s002.docx]

**Supporting File 2: Survey questionnaire and qualitative question guide, AE study**

| **Survey Questionnaire** | |
| --- | --- |
|  | I believe it is important to take deworming tablets for my health. To what extent do you agree/disagree with this statement? |
|  | I believe taking deworming tablets is necessary for people who do not have symptoms of worm infections. To what extent do you agree/disagree with this statement? |
|  | I believe taking deworming tablets is not necessary for people who always uses toilet. To what extent do you agree/disagree with this statement? |
|  | I believe that STH are minor infections that cause little harm to the body. To what extent do you agree/disagree with this statement? |
|  | I think deworming tablet is a safe drug. To what extent do you agree/disagree with this statement? |
|  | I think the deworming tablets distributed can cause adverse events. To what extent do you agree/disagree with this statement? |
|  | I think deworming tablet is an effective drug. To what extent do you agree/disagree with this statement? |
|  | STH mainly affects children and not adults. To what extent do you agree or disagree with this statement? |
|  | Most of my neighbours and friends eat the deworming tablet during deworming campaigns. To what extent do you agree/disagree with this statement? |
|  | Most of my neighbours and friends will expect me/my child to eat the deworming tablet during distribution campaigns. To what extent do you agree/disagree with this statement? |
|  | People in my village think that it is important to control or stop the spread of worms. To what extent do you agree/disagree with this statement? |
|  | The participation or lack of participation of my neighbours during deworming influences my decision to participate. To what extent do you agree/disagree with this statement? |
|  | In future, if this deworming tablet is distributed to everyone, I would eat this tablet (or give to my child), even if my family members tell me not to. To what extent do you agree/disagree with this statement? |
|  | I am confident that I can access deworming medications during campaigns (when distributed to everyone) if I want them. To what extent do you agree/disagree with this statement? |
|  | In future, I would continue to eat the deworming tablet which is distributed to everyone (or given to my child), even if neighbours or friends tell me not to. Do you agree/disagree with this statement? |
|  | I do not need the approval of my family members before taking deworming tablet or giving them to my children. To what extent do you agree/disagree with this statement? |
|  | Who is the main person in your family who decided that everyone in your family should take the deworming tablet distributed to everyone in your community? |
|  | How expensive or inexpensive do you think it is to purchase deworming tablet from a medical store to deworm everyone in your family? |
| 1. 1 | I worry about adverse effects for myself during future distributions of deworming tablets. To what extent do you agree/disagree with this statement? |
| 1. 2 | I worry about adverse effects for my family members during future distributions of deworming tablets. To what extent do you agree/disagree with this statement? |
| 1. 3 | The experience of adverse effect of this deworming tablet is likely to influence my decision to eat (or allow my child to eat) deworming tablets if distributed in future. To what extent do you agree/disagree with this statement? |
| 1. 4 | I am planning to participate (or allow my child to participate) in the next round of deworming in my community. Do you agree/disagree with this statement? |

**Qualitative question guide**

1. You/your child had experienced adverse effect after taking the deworming tablet that was distributed to everyone in the village. Please tell me about that experience––what were the adverse effects and what happened. (Probe: Get details about all that happened until the resolution of the adverse effects with a timeline. Probe for possible reasons for the adverse effect)
2. What is your opinion about the deworming tablet that was distributed to you/your child? (Probe: why good/bad or why any advantage/disadvantage) When you/your child had the adverse effect, what did you think about the tablet?
3. What do your family members says about the tablet? Probe: who said what and why) What did your family members say when you/your child experienced the adverse effects? (Probe: who said what and why)
4. Among your family members, whose suggestions or advise is important for you or your child to take/not to take the deworming tablets that is distributed to everyone and why?
5. What do your neighbours and friends says about the tablet? (Probe: who said what and why)What did your neighbours and friends say when you/your child experienced the adverse effects? (Probe: who said what and why)
6. How important is your neighbours and friends opinion for you/your child to take/not take this deworming tablets that is distributed to everyone and why?
7. Did anyone from deworm 3 team visit you when you/your child experienced adverse effect? What did they say? To what extent you were satisfied/not satisfied with the treatment/advise and why? What doubt or questions about the tablet and adverse effects did you or your family members have even after they spoke to you?
8. What did other healthcare providers like the village nurse or any doctor or traditional healer say when you/your child had the adverse effect? What did they say about the tablet and why?
9. In your opinion, what should have been done when you/your child experienced adverse effect and why?
10. After the adverse event, the next time when the tablet was distributed, did you/your child eat the tablet? Why? (Probe: who said what in the decision making­­­–– family members/neighbours/friends/DeWorm3 staff/anyone else) How did you feel about eating that tablet [ or giving that tablet to your child] again and why?
11. Have you or your family members ever taken deworming tablet other than what was distributed by DeWorm3? Who all took, when did they take, from where, what happened after eating that tablet?
12. How important is it for everyone in your family to take the deworming tablet at the same time and why? (Probe: Do you think, you or your family members probably have intestinal worms, why?)
13. If the distribution of deworming tablet is stopped, what will you do and why? (Probe: What helps/does not help for everyone in the family to eat?)
14. Do you think it is important/not important for everyone in your village to take the deworming tablet at the same time and why?
