# Supplementary material for: Impact of adverse events during community-wide mass drug administration for soil-transmitted helminths on subsequent participation–a Theory of Planned Behaviour analysis
Source: PLoS Negl Trop Dis. 2023 Mar 14;17(3):e0011148. doi: 10.1371/journal.pntd.0011148 (PMC10013912; doi:10.1371/journal.pntd.0011148)
Supplement: Attachment — Submitted filename: PNTD-D-22-01128_reviewer.pdf [file pntd.0011148.s003.pdf]

PLOS Neglected Tropical Diseases

Impact of adverse events during community-wide mass drug administration for soil-transmitted helminths on subsequent participation – a Theory of Planned Behaviour analysis

--Manuscript Draft--

**Manuscript Number:**	PNTD-D-22-01128
**Full Title:**	Impact of adverse events during community-wide mass drug administration for soil-transmitted helminths on subsequent participation – a Theory of Planned Behaviour analysis
**Short Title:**	Adverse events in cMDA for STH
**Article Type:**	Research Article
**Keywords:**	adverse events; Soil-Transmitted Helminths; mass drug administration; albendazole; theory of planned behaviour; DeWorm3
**Abstract:**	Background: Experiencing adverse events (AEs) during mass drug administration (MDA) could affect participation in future MDAs. This study aims to understand the potential influence of AEs during a community-wide MDA (cMDA) trial for soil-transmitted helminths in India on intention to participate in future cMDAs.<br>Methods: This study was conducted using a multi-method quantitative and qualitative approach among those who had experienced an AE and subsequently refused cMDA treatment. Path analysis and thematic analysis, guided by the Theory of Planned Behaviour, was used.<br>Principal findings: Among 74 individuals with an identified AE, 12% refused treatment in the cMDA immediately subsequent to their AE and 4% refused in all subsequent cMDAs. Of these 74 individuals, 59 (80%) completed a survey and eight participated in in-depth interviews. A positive attitude towards deworming and perceived ability to participate in cMDA (perceived behavioural control) were significant predictors of intention to participate in cMDA ($p<0.05$). A positive attitude towards cMDA was associated with caste ($\chi^2=3.83$, $P=0.05$), particularly among the scheduled caste/scheduled tribe (SC/ST) (62%). Perceived behavioural control in cMDA participation was associated with occupation ($\chi^2=5.02$, $P<0.05$), with higher perceived control among those engaged in skilled occupations (78%). Intention to participate in subsequent cMDAs was associated with caste and family type ($\chi^2=3.83$, $P=0.05$ and $\chi^2=7.50$, $P<0.05$ respectively) and was higher among SC/ST (62%) and those with extended families (67%). In-depth interviews demonstrated that perceived severe AEs may lead to treatment refusal, particularly if children were affected.<br>Conclusions: Attitudes towards cMDA and perceived behavioural control in cMDA participation are significantly associated with the intention to participate in future cMDA after experiencing an AE. Community mobilization messages about potential AEs and their management may need to intentionally target non-SC/ST households, nuclear families, and those engaged in unskilled occupations to increase cMDA participation given the possibility of AEs occurring.
**Additional Information:**	
**Question**	**Response**
**Financial Disclosure**<br><br>Enter a financial disclosure statement that describes the sources of funding for the work included in this submission. Review the submission guidelines for detailed requirements. View published research articles from *PLOS NTDs* for specific examples.	The DeWorm3 Project is funded by a grant from the Bill & Melinda Gates Foundation (OPP1129535, PI JLW). The funders had no role in study design, data collection, analysis, decision to publish, or manuscript preparation.

Powered by Editorial Manager® and ProduXion Manager® from Aries Systems Corporation

This statement is required for submission and **will appear in the published article** if the submission is accepted. Please make sure it is accurate.<br><br>**Unfunded studies**<br><br>Enter: *The author(s) received no specific funding for this work.*<br><br>**Funded studies**<br><br>Enter a statement with the following details:<br>• Initials of the authors who received each award<br>• Grant numbers awarded to each author<br>• The full name of each funder<br>• URL of each funder website<br>• Did the sponsors or funders play any role in the study design, data collection and analysis, decision to publish, or preparation of the manuscript?<br>• **NO** - Include this sentence at the end of your statement: *The funders had no role in study design, data collection and analysis, decision to publish, or preparation of the manuscript.*<br>• **YES** - Specify the role(s) played.<br><br>* typeset	
**Competing Interests**<br><br>Use the instructions below to enter a competing interest statement for this submission. On behalf of all authors, disclose any competing interests that could be perceived to bias this work—acknowledging all financial support and any other relevant financial or non-financial competing interests.<br><br>This statement **will appear in the**	The authors have declared that no competing interests exist.

Powered by Editorial Manager® and ProduXion Manager® from Aries Systems Corporation

**published article** if the submission is accepted. Please make sure it is accurate. View published research articles from *PLOS NTDs* for specific examples.<br><br>**NO authors have competing interests**<br><br>Enter: *The authors have declared that no competing interests exist.*<br><br>**Authors with competing interests**<br><br>Enter competing interest details beginning with this statement:<br><br>*I have read the journal's policy and the authors of this manuscript have the following competing interests: [insert competing interests here]*<br><br>* typeset<br><br>This statement is required for submission and **will appear in the published article** if the submission is accepted. Please make sure it is accurate and that any funding sources listed in your Funding Information later in the submission form are also declared in your Financial Disclosure statement.	
**Data Availability**<br><br>Authors are required to make all data underlying the findings described fully	No - some restrictions will apply

Powered by Editorial Manager® and ProduXion Manager® from Aries Systems Corporation

available, without restriction, and from the time of publication. PLOS allows rare exceptions to address legal and ethical concerns. See the PLOS Data Policy and FAQ for detailed information.<br><br>A Data Availability Statement describing where the data can be found is required at submission. Your answers to this question constitute the Data Availability Statement and **will be published in the article**, if accepted.<br><br>**Important:** Stating 'data available on request from the author' is not sufficient. If your data are only available upon request, select 'No' for the first question and explain your exceptional situation in the text box.<br><br>Do the authors confirm that all data underlying the findings described in their manuscript are fully available without restriction?	
**Describe where the data may be found in full sentences. If you are copying our sample text, replace any instances of XXX with the appropriate details.**<br><br>• If the data are **held or will be held in a public repository**, include URLs, accession numbers or DOIs. If this information will only be available after acceptance, indicate this by ticking the box below. For example: *All XXX files are available from the XXX database (accession number(s) XXX, XXX.*).<br>• If the data are all contained **within the manuscript and/or Supporting Information files**, enter the following: *All relevant data are within the manuscript and its Supporting*	All relevant data are within the manuscript and its supporting information files.

Information files.<br>• If neither of these applies but you are able to provide **details of access elsewhere**, with or without limitations, please do so. For example:<br><br>*Data cannot be shared publicly because of [XXX]. Data are available from the XXX Institutional Data Access / Ethics Committee (contact via XXX) for researchers who meet the criteria for access to confidential data.*<br><br>*The data underlying the results presented in the study are available from (include the name of the third party and contact information or URL).*<br>• This text is appropriate if the data are owned by a third party and authors do not have permission to share the data.<br><br>* typeset	
Additional data availability information:	

Manuscript Click here to access/download;Manuscript;Manuscript.docx

Impact of adverse events during community-wide mass drug administration for soil-transmitted helminths on subsequent participation – a Theory of Planned Behaviour analysis

Authors

Kumudha Aruldas[1,¶], Gideon John Israel[1,¶], Jabaselvi Johnson[1], Angelin Titus[1], Malvika Saxena[1], Saravanakumar Puthupalayam Kaliappan[1], Rohan Michael Ramesh[1], Judd L. Walson[2,4], Arianna Rubin Means[3,4], and Sitara SR Ajjampur[1*]

Affiliations

[1]The Wellcome Trust Research Laboratory, Division of Gastrointestinal Sciences, Christian Medical College, Vellore, India

[2]Departments of Global Health, Medicine, Pediatrics and Epidemiology, University of Washington, Seattle, Washington, USA

[3]Department of Global Health, University of Washington, Seattle, USA

[4]The DeWorm3 Project, University of Washington, Seattle, Washington, USA

¶ Co-lead authors

*** Correspondence:** Sitara Swarna Rao Ajjampur

sitararao@cmcvellore.ac.in

ORCID number: 0000-0003-3419-6577

Short title

Adverse events in cMDA for STH

Abstract

Background: Experiencing adverse events (AEs) during mass drug administration (MDA) could affect participation in future MDAs. This study aims to understand the potential influence of AEs during a community-wide MDA (cMDA) trial for soil-transmitted helminths in India on intention to participate in future cMDAs.

Methods: This study was conducted using a multi-method quantitative and qualitative approach among those who had experienced an AE and subsequently refused cMDA treatment. Path analysis and thematic analysis, guided by the Theory of Planned Behaviour, was used.

Principal findings: Among 74 individuals with an identified AE, 12% refused treatment in the cMDA immediately subsequent to their AE and 4% refused in all subsequent cMDAs. Of these 74 individuals, 59 (80%) completed a survey and eight participated in in-depth interviews. A positive attitude towards deworming and perceived ability to participate in cMDA (perceived behavioural control) were significant predictors of intention to participate in cMDA ($p<0.05$). A positive attitude towards cMDA was associated with caste (χ^2=3.83, P=0.05), particularly among the scheduled caste/scheduled tribe (SC/ST) (62%). Perceived behavioural control in cMDA participation was associated with occupation (χ^2=5.02, P<0.05), with higher perceived control among those engaged in skilled occupations (78%). Intention to participate in subsequent cMDAs was associated with caste and family type (χ^2=3.83, P=0.05 and χ^2=7.50, P<0.05 respectively) and was higher among SC/ST (62%) and those with extended families (67%). In-depth interviews demonstrated that perceived severe AEs may lead to treatment refusal, particularly if children were affected.

Conclusions: Attitudes towards cMDA and perceived behavioural control in cMDA participation are significantly associated with the intention to participate in future cMDA after experiencing an AE. Community mobilization messages about potential AEs and their management may need to intentionally target non-SC/ST households, nuclear families, and

those engaged in unskilled occupations to increase cMDA participation given the possibility of AEs occurring.

Keywords: adverse events, soil-transmitted helminths, mass drug administration, albendazole, theory of planned behaviour, DeWorm3

Author summary

This paper sampled individuals who experienced an AE during community-wide MDA (cMDA) with albendazole for soil-transmitted helminths in the DeWorm3 trial in India to understand their intention to participate in future cMDAs. This study utilized the theory of planned behaviour, which postulates that an individual's intention to change a behaviour depends on their attitude, perceived expectations of people around them (social norms), and their perceived behavioural control to adopt the behaviour (self-efficacy). This study indicated that positive attitude towards cMDA and their perceived behavioural control in cMDA participation positively influences their intention to participate in future cMDAs. Other factors associated with intention to participate in cMDA were caste, family structure, occupation, and participation in community sensitisation activities. In-depth interviews revealed that while many individuals participate in subsequent cMDAs after an AE because of the presumed health benefits of deworming, some are likely to refuse treatment due to fear of AEs, particularly fears of AEs among children. In order to increase participation rates in cMDA programs where AEs undoubtably do occur, targeted counselling of groups at high risk of non-participation and assuring care and support during AEs may be important strategies to improve coverage.

Introduction

India is estimated to have about 258 million people infected with soil-transmitted helminths (STH) including *Ascaris lumbricoides*, *Trichuris trichiura*, and *Ancylostoma duodenale* or *Necator americanus* (hookworms) (1). The World Health Organization (WHO) Roadmap 2030 for Neglected Tropical Diseases (NTD) includes the goal to eliminate STH-related morbidity (usually caused by moderate to heavy intensity infections, MHI) by ensuring 75% coverage in targeted deworming of vulnerable populations including pre-school aged children (PSAC) 1–4 years), school-age children (SAC) (5–19 years) and women of reproductive age (WRA) (2). Mathematical modelling and the 'TUMIKIA' field trials indicate that expanded, community-wide deworming with mass drug administration (cMDA) could potentially interrupt transmission of STH (3-6). In this context, a multi-country community-based cluster-randomized trial, DeWorm3, is underway in Benin, India, and Malawi to assess the feasibility of interrupting STH transmission with six rounds of biannual cMDA of albendazole (7-9).

Adverse events (AEs) experienced during treatment in MDAs and the fear of side effects to the treatment during MDAs can often lead to refusals to participate in MDA programs (10-14). Further, not receiving care for suspected AEs previously experienced during MDA can also deter community members from participating in future MDAs (15). In India, albendazole has been co-administered with diethylcarbamazine in Lymphatic Filariasis (LF) MDA programs since 2007. Albendazole is also administered biannually in the country's school-based deworming program, one of the largest in the world targeting ~240 million children (16,17). Several LF-MDA studies in India have reported AEs in the range of 3-9% such as dizziness, drowsiness, nausea, vomiting, and fever, however, they are not specific to albendazole intake alone (10,11,13,18,19). There are limited data concerning participant behaviours after an AE, and how experiencing an AE influences willingness to participate in future rounds of cMDA.

The Theory of Planned Behaviour (TPB) describes three aspects of human intention to adopt a behaviour or action, such as participating in cMDA after experiencing an AE (20). One is participant's attitude, the individual's beliefs about the expected positive or negative outcomes of the behaviour or action; second is subjective norms, an individual's perceived social or peer expectations of their behaviour or action; and third is perceived behavioural control, the individual's beliefs about their ability or self-efficacy to adopt the behaviour or perform the action. These individual characteristics influence intention to adopt a future behaviour or take a future action, such as participation in deworming programs.

Very few studies have used the TPB to assess participation or uptake of interventions for infectious diseases. These include assessment of interventions such as sleeping under an insecticide-treated mosquito net for malaria prevention in Iran and adherence to tuberculosis treatment in Indonesia (21,22). However, several studies have used the TPB to evaluate intentions to adopt COVID-19 related behaviour changes as preventive measures during the pandemic, such as maintaining physical distancing, wearing a mask, and vaccination uptake in a range of countries including the USA, China, Pakistan, and Israel (23-28). This multi-method study aimed to use the TPB framework to understand perceptions about participating in cMDA for STH and drivers of participation in subsequent STH cMDA programs after experiencing an AE.

Methods

Study location and setting

The DeWorm3 trial site in southern India is located in two blocks of Tamil Nadu - the Timiri block in Ranipet district (formerly Vellore district) and the Jawadhu Hills block in Tiruvannamalai district. At the start of the trial in 2017, 140,932 individuals were enumerated in the study area residing in 6,536 households across 373 villages. The socio-demographic details of the study population and the baseline STH prevalence have been previously published (7,29). The population was demarcated into 40 clusters and was randomized to 20 intervention and 20 control clusters. (8) During the cMDA in the intervention clusters, the community drug distributors (CDDs) distributed albendazole door-to-door to all community members from the ages of 1–99 years. In the control clusters, the Ministry of Health and Family Welfare (MoHFW) implemented routine biannual school-based treatment of PSAC and SAC on National Deworming Days (16). During cMDA, field workers and supervisors engaged in the trial, accompanied the CDDs to capture real-time treatment consumption data using a mobile phone application. The study was initiated in 2017, completed six rounds of biannual cMDA during 2018-2020 and is currently in the post-intervention surveillance phase.

During and for seven days following the cMDAs, passive surveillance of AEs was conducted where community members reported their AEs to the CDDs or the field workers. The CDDs informed the field workers and they in turn reported the AEs immediately to the respective medical teams set up for the trial in the two study blocks. The medical teams visited households where an AE was reported, documented the details of the AE, and provided treatment, follow-up care, and support.

Study design and data collection

A multi-method approach was used, including quantitative and qualitative data collection. The medical teams recorded 75 AEs among 74 participants (one participant reported AEs in two

rounds of cMDA) during the six rounds of cMDA. All 74 individuals who reported experiencing an AE were invited to complete a survey during March-April 2021, six months after completing the last cMDA. For AEs reported among the 14 children under 15 years, the primary caretaker present at the time of their AE participated in the survey on their behalf. In parallel, eight in-depth interviews were conducted with individuals who reported a prior AE and also refused treatment in one or more of the subsequent cMDA rounds. Primary caregivers of children (two out of eight) were interviewed on behalf of the children.

The participation in STH cMDA is a voluntary preventive health behaviour, therefore, the TPB constructs were used to guide the development of the questionnaire, qualitative guidelines, and data analysis (20). Quantitative data were collected via a 37-item questionnaire consisting of 15 questions about independent variables, 19 questions with 5-point Likert scale questions ('strongly agree' to 'strongly disagree') and three questions with 3-category nominal scale questions ('yes', 'not sure', and 'no') to measure the outcome variables (S1 file). The questionnaire was translated into Tamil (local language), piloted, and administered by four trained research assistants.

The in-depth interviews were conducted using a semi-structured guideline (S1 file) to understand participant's experiences of an AE, why they declined subsequent treatment and their perspective on participating in future STH cMDA programs. The in-depth interviews were conducted in the local language (Tamil) by two trained social science research assistants using an interview guide and were audio-recorded.

Data analysis

We hypothesized that individuals with favourable beliefs towards participating in cMDA would have a stronger intention to participate in future cMDA rounds, even after reporting an AE. The responses to five-point Likert scale questions of ‘strongly agree’ to ‘strongly disagree’ were scored as 5 to 1, with 5 being the most positive response and 1 being the most negative response. The responses to the three category nominal scale questions of ‘yes’, ‘not sure’, and ‘no’, were scored as 5, 3, and 1, respectively. All questions were given equal weightage. The individual scores of the questions in each TPB construct were added to arrive at the composite score. The scores equal to and above the median of the composite scores were categorised as a ‘high’ or ‘positive’ construct and below-median as a ‘low’ or ‘negative’ construct (30).

The internal consistency of the questionnaire was tested using Cronbach’s alpha for reliability (31). The questions for measurement of attitude (eight questions), perceived behavioural control (six questions), subjective norm (four questions), and intention to participate in future cMDAs (four questions) had an alpha value of 0.65, 0.64, 0.44 and 0.74, respectively. The 15 independent variables were respondent age, gender, marital status, caste, religion, education, occupation, family structure, highest level of household education and occupation, household toilet, membership in any community-based organizations, exposure to community sensitization activities, familiarity with the CDD carrying out cMDA in their neighbourhood, and perceived severity of AEs. Descriptive statistics were used to report the total number and proportion of individuals in each demographic group. Chi-Square and Fisher’s exact test, significant at $P<0.05$, was used to determine if there are associations between the independent variables of interest and the four outcome variables. We used multiple regression-based path analysis to examine the relationship between a dependent variable and two or more independent variables to evaluate causal models (32). This determines the degree and significance of the causal relationship between variables. The path analysis calculates all the

paths simultaneously and yields overall goodness of fit measure for the model. In this analysis, the structural path model, a type of directed graph, was formed by linking the independent variables (attitude, subjective norm, and perceived behavioural control) to the outcome variable (intention to participate), where each independent variable is hypothesized to influence the outcome variable. The data were analysed with STATA 16.0 software (StataCorp, TX, USA) and GPower 3.1 was used to calculate the power of a statistical test. The audio-recorded in-depth interviews were transcribed verbatim and translated into English by the two interview facilitators. The transcripts were coded by two primary coders independently using a deductive approach using an *a priori* TPB-based codebook, final codes arrived with consensus, and analysed in ATLAS.Ti 8.0.

Ethics statement

The Institutional Review Board (IRB) of Christian Medical College (CMC), Vellore (10392 [INTERVEN]), and the Human Subjects Division at the University of Washington (STUDY00000180) approved the DeWorm3 trial in India. The trial is registered at ClinicalTrials.gov (NCT03014167). The CMC IRB approved this study on AE among Deworm3 trial participants as an amendment (IRB – A12, October 28, 2020). Written informed consent was obtained from all who participated in the survey and in-depth interviews.

Results

Experience of AEs and participation in subsequent cMDAs

A total of 74 participants reported related or unrelated AEs during the six rounds of cMDA, and of these, three (one child and two adult women) were reported determined a serious AE by the medical team but unrelated to albendazole. Overall, 8% of the AEs recorded were in PSAC,

14% were in SAC, 22% were in men, and 57% were in women above the age of 15 years. The AEs recorded by the medical teams based on the complaints reported by the participants were diarrhoea (49, 66.2%), gastritis (8, 10.8%), skin allergy (6, 8.1%), fever (5, 6.8%), stomach pain (3, 4.1%), cough (1, 1.4%), dizziness (1, 1.4%), and urinary tract infection (1, 1.4%).

Fifty-nine of the 74 eligible participants consented to participate in the survey on AE (80%) (Figure 1). Of the 59 survey participants, 43 were women (73%), and 16 were men (27%). They were between the ages of 19–72 years (median, interquartile range (IQR): 45.0, 29.0-56.0). A majority were Hindu (93%), many were married (75%), and a little over half belonged to a disadvantaged socio-economic group listed in a schedule of the Indian constitution as scheduled caste or scheduled tribe (58%) (SC/ST), and lived in an extended family (includes siblings and their families or has at least three generations) (56%). Less than half were educated to the level of secondary school or more (44%), however, about 86% had someone in their family who was educated to the level of secondary school or more. Among the participants, 44% had someone in the family who was a member of either a women's self-help group, a farmers' club, or a cooperative society. About half of them had attended DeWorm3 community sensitization activities conducted prior to a cMDA (49%), and many were familiar with the CDDs delivering treatment in their village (88%). Of the 15 individuals who did not participate in the survey, 2 (13%) were children aged 1-15 years, 8 (54%) were women, and 5 (33%) were men for reasons such as they were fine so did not feel the need to participate.

Fig 1: AE study participation chart

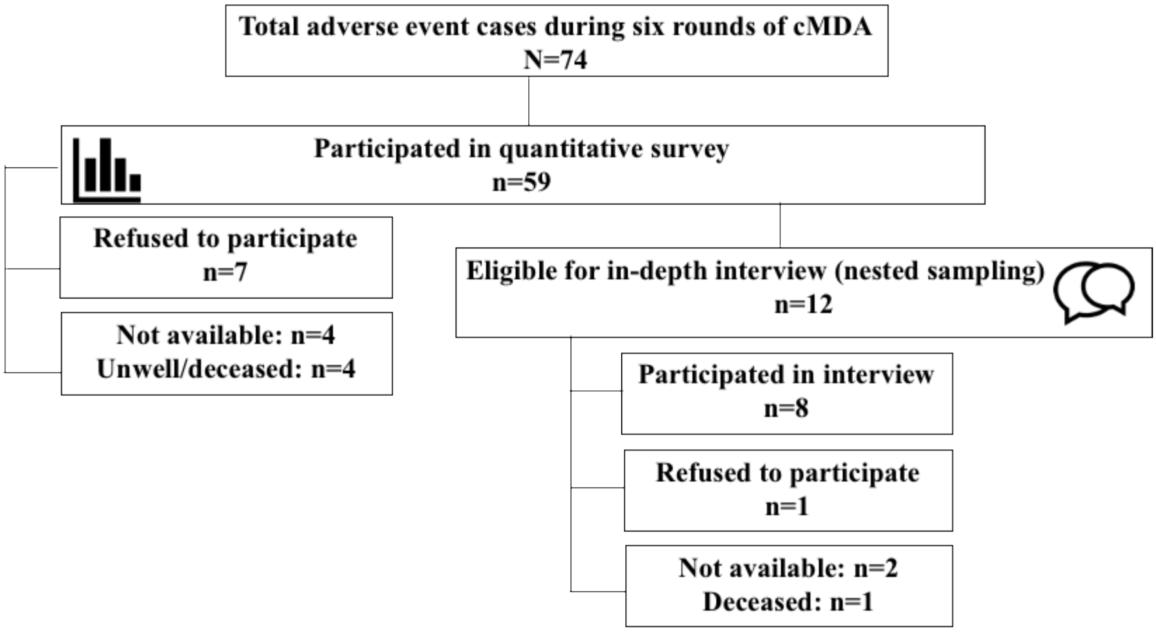

Of the 74 who experienced an AE, 84% consumed the tablet in all subsequent rounds of cMDA, 12% refused treatment in the immediate subsequent round, and 4% refused treatment in all subsequent rounds (Figure 2). Among the 12 who refused to treatment in one or more subsequent rounds of cMDA, eight out of the nine participants consented to an in-depth interview. The one who did not participate was an elderly man and the family members did want him to be disturbed him with an interview.

Fig 2: Treatment history of 12 participants who refused treatment in subsequent rounds of cMDA after experiencing an AE

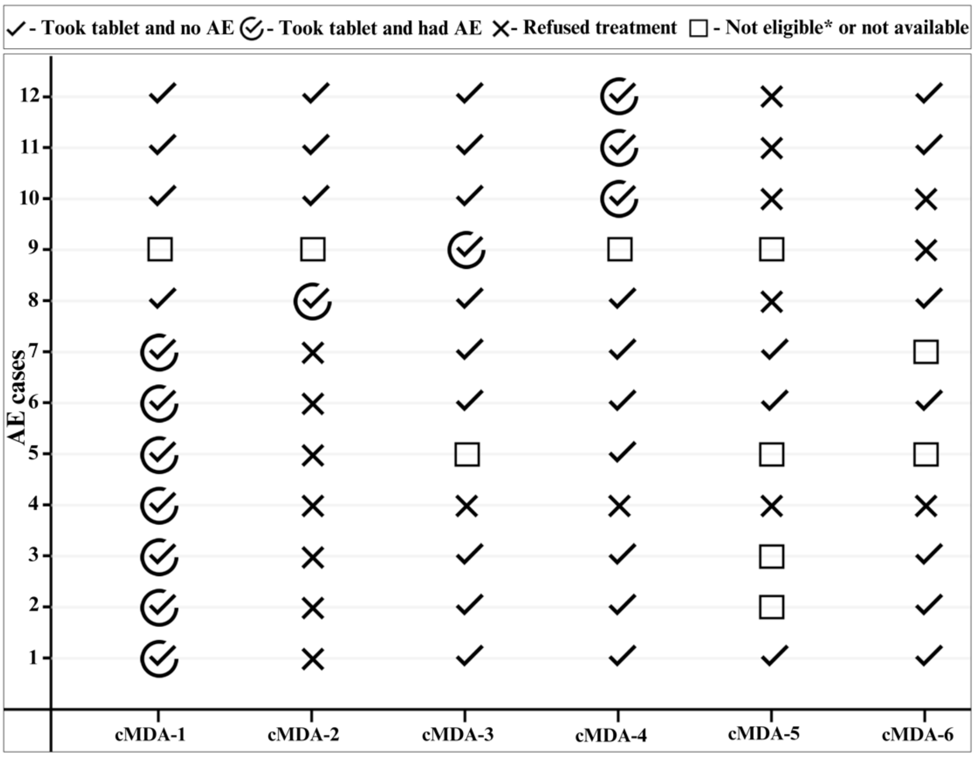

* Not eligible - seriously ill or received deworm treatment within two weeks prior to cMDA

CDDs were the first person most participants contacted when they experienced an AE, and were satisfied with their response

Most of the participants were familiar with the CDDs who distributed the albendazole to them during the cMDAs (88%). CDDs were the first person the participants contacted when they experienced an AE (81%) and were 'very satisfied or satisfied' with the advice given and actions taken by the CDD (94%). As a next step, the CDDs informed the DeWorm3 field workers about the AEs and coordinated the house visits of medical teams. The in-depth interviews also revealed that most respondents were familiar with the CDDs and some even knew them by their names because the CDDs were from the same village. The participants contacted the CDDs immediately if the symptoms occurred on the same day after treatment and when they perceived that the symptom was due to the tablet. Those who did not attribute the cause of the symptom to the treatment waited for two days before informing the CDDs.

The participants were satisfied with CDDs because they had visited them immediately after the AE was reported and subsequently initiated house visits and follow-ups of medical teams. They said,

> "*She (CDD) came immediately from next street, within 5 minutes… she called the doctor on phone…. Doctor also came…. kept coming for three days... and spoke well. Doctor asked me to call immediately if something happens, and they will come immediately… Yes, I was satisfied.*" (Woman #1- perceived moderately severe AE, Cluster 23)

> "*I went to see her (CDD) and asked her, "What tablet did you give me?... Immediately, she called those who came along with her to distribute the tablet. They came immediately… doctor was one among them, they gave me a tablet, after eating that tablet I got well… To a great extent I am satisfied… Is it not good when things happen in a good manner?*" (Man #1- perceived severe AE, Cluster 13)

Despite experiencing an AE, most believed that the tablet was safe and effective, and deworming was necessary

Among the 59 participants surveyed, 58% perceived their AE to be 'severe or very severe', 24% reported 'moderate' severity and 18% reported 'mild or very mild' severity. Most of the participants attributed their AE to multiple causes, including albendazole distributed during cMDA (85%), worms they had in their stomach (67%), food they had consumed that day (22%), other associated illnesses (5%), or to consuming the tablet on an empty stomach (4%). The in-depth interviews also showed that some of them strongly believed that the AE occurred directly and solely due to the tablet, as they said,

"*Yes, only due to that tablet…I had suffered that sort of diarrhoea,… it happened because of eating that tablet…. I did not have any other problem, it happened by eating the tablet.*" (Woman #1- perceived moderately severe AE, Cluster 24)

"*I ate only dhal and rice on that day, which is usually what I eat. For indigestion to happen, did I eat meat and rice, to cause it? Only on that day… This happened only after eating this tablet.*" (Man #1- perceived severe AE, Cluster 13)

Despite attributing the AE to the tablet distributed during cMDAs, a majority indicated that deworming was good for their health (93%), and the drug distributed in cMDAs was safe and effective (85%). Most of them believed that those who do not have symptoms of STH should also be dewormed (88%) but about half of them thought that deworming was not necessary for those who use toilets (53%). Many of them believed that STH is only a minor infection (49%) but believed that the adults were also affected (80%). During the in-depth interviews, they explained this as,

"*Everyone has intestinal worms. It is not that intestinal worms affect only adults or children, but intestinal worms do exist in excess for children… Intestinal worms come out automatically when adults eat tablet for purging and cleansing… We do not know whether in our stomach intestinal worms exist or not.*" (Man #1- perceived severe AE, Cluster 13)

"*It (STH) is not a minor one… It will become a lump… cause trouble to the body, every month menstruation will not be regular,… if we eat the tablet, menstruation happens correctly… Infection can start with adults later transmit to children as well.*" (Woman #1- perceived moderately severe AE, Cluster 23)

In-depth interviews among those who had refused to participate in at least one cMDA, showed that they perceived their AE to be mild to moderately severe. Most of them reported that their symptoms appeared within a few hours of ingesting the tablet and lasted for about 1-3 days.

Fear of an AE was also observed irrespective of their perceived severity of an AE. Fear of severe AEs among children can prevent them from participating in future cMDAs. They said,

> "*I am eating the tablet which drug distributors give…. I strongly believe it is important for me. I tell my family members to eat. After my grandson's sickness which happened after he ate the tablet, I cannot force my family to eat the tablet. I think eating deworm tablet is important.*" (Grandfather- perceived very severe AE, Cluster 15)

> "*Six months ago, they gave the tablet and I had diarrhoea. When they came again to give the tablet, I said no to tablet, rest of my family members also did not eat the tablet. No one ate the tablet. Same fear.*" (Man #1- perceived severe AE, Cluster 13)

Overall, 51% of individuals had a positive attitude (≥median score of 34; maximum score–40) towards cMDA, despite having reported a previous AE. Positive attitude towards STH cMDA was significantly associated with caste (χ^2=3.83, P=0.05) (Table 1). A higher proportion of those who belonged to SC/ST caste (62%) had a positive attitude towards cMDA for STH than those who belonged to other castes (36%).

Table 1. Factors associated with attitude, subjective norm, perceived behavioural control, and intention to participate in future STH cMDAs

Factors	Total	Attitude			Subjective norm			Perceived behavioural control			Intention to participate		
	n=59	Low n=29	High n=30	Chi-Square test	Low n=29	High n=30	Chi-Square test	Low n=26	High n=33	Chi-Square test	Low n=29	High n=30	Chi-Square test
	n (%)	n (%)	n (%)	p-value	n (%)	n (%)	p-value	n (%)	n (%)	p-value	n (%)	n (%)	p-value
**Gender**													
Male	16 (27.12)	11 (68.75)	5 (31.25)	**0.066**	8 (50.00)	8 (50.00)	0.937	9 (56.25)	7 (43.75)	0.250	10 (62.50)	6 (37.50)	0.211
Female	43 (72.88)	18 (41.86)	25 (58.14)		21 (48.84)	22 (51.16)		17 (39.53)	26 (60.47)		19 (44.19)	24 (55.81)	
**Age**													
Below median (<45 years)	28 (47.46)	12 (42.86)	16 (57.14)	0.358	16 (57.14)	12 (42.86)	0.243	13 (46.43)	15 (53.57)	0.728	12 (42.86)	16 (57.14)	0.358
Above median	31 (52.54)	17 (54.84)	14 (45.16)		13 (41.94)	18 (58.06)		13 (41.94)	18 (58.06)		17 (54.84)	14 (45.16)	
**Marital status**													
Married	44 (74.58)	19 (43.18)	25 (56.82)	0.116	19 (43.18)	25 (56.82)	0.116	18 (40.91)	26 (59.09)	0.403	20 (45.45)	24 (54.55)	0.33
Others [a]	15 (25.42)	10 (66.67)	5 (33.33)		10 (66.67)	5 (33.33)		8 (53.33)	7 (46.67)		9 (60.00)	6 (40.00)	
**Education**													
Below secondary	39 (66.10)	22 (56.41)	17 (43.59)	0.119	19 (48.72)	20 (51.28)	0.926	19 (48.72)	20 (51.28)	0.315	21 (53.85)	18 (46.15)	0.314
Secondary and above	20 (33.90)	7 (35.00)	13 (65.00)		10 (50.00)	10 (50.00)		7 (35.00)	13 (65.00)		8 (40.00)	12 (60.00)	
**Highest level of household education**													
Below secondary	12 (20.34)	6 (50.00)	6 (50.00)	0.948	7 (58.33)	5 (41.67)	0.476	5 (41.67)	7 (58.33)	1.000 [b]	3 (25.00)	9 (75.00)	**0.061**
Secondary and above	47 (79.66)	23 (48.94)	24 (51.06)		22 (46.81)	25 (53.19)		21 (44.68)	26 (55.32)		26 (55.32)	21 (44.68)	
**Religion**													
Hindu	55 (93.22)	27 (49.09)	28 (50.91)	1.000 [b]	27 (49.09)	28 (50.91)	1.000 [b]	24 (43.64)	31 (56.36)	0.805	28 (50.91)	27 (49.06)	0.612 [b]
Others	4 (6.78)	2 (50.00)	2 (50.00)		2 (50.00)	2 (50.00)		2 (50.00)	2 (50.00)		1 (25.00)	3 (75.00)	
**Caste**													
SC/ST	34 (57.63)	13 (38.24)	21 (61.76)	**0.050** [c]	15 (44.12)	19 (55.88)	0.367	12 (35.29)	22 (64.71)	0.113	13 (38.24)	21 (61.76)	**0.050** [c]
Others (MBC/BC/FC)	25 (42.37)	16 (64.00)	9 (36.00)		14 (56.00)	11 (44.00)		14 (56.00)	11 (44.00)		16 (64.00)	9 (36.00)	

**Family type**													
Nuclear family	26 (44.07)	14 (53.85)	12 (46.15)	0.522	18 (69.23)	8 (30.77)	**0.006** [c]	13 (50.00)	13 (50.00)	0.415	18 (69.23)	8 (30.77)	**0.006** [c]
Extended family	33 (55.93)	15 (45.45)	18 (54.55)		11 (33.33)	22 (66.67)		13 (39.39)	20 (60.61)		11 (33.33)	22 (66.67)	
**Occupation**													
Unskilled/Semi-skilled	37 (67.27)	21 (51.22)	20 (48.78)	0.504	20 (48.78)	21 (51.22)	0.931	22 (53.66)	19 (46.34)	**0.025** [c]	19 (46.34)	22 (53.66)	0.391
Skilled	18 (32.73)	8 (44.44)	10 (55.56)		9 (50.00)	9 (50.00)		4 (22.22)	14 (77.78)		10 (55.56)	8 (44.44)	
**Highest level of household occupation**													
Unskilled/Semi-skilled	48 (87.27)	23 (44.23)	29 (55.77)	0.101 [b]	25 (48.08)	27 (51.92)	0.706 [b]	24 (46.15)	28 (53.85)	0.449 [b]	25 (48.08)	27 (51.92)	0.696 [b]
Skilled	7 (12.73)	6 (85.71)	1 (14.29)		4 (57.14)	3 (42.86)		2 (28.57)	5 (71.43)		4 (57.14)	3 (42.86)	
**Household toilet**													
No	34 (57.63)	17 (50.00)	17 (50.00)	0.879	17 (51.52)	16 (48.48)	0.683	17 (50.00)	17 (50.00)	0.284	20 (58.82)	14 (41.18)	**0.083**
Yes	25 (42.37)	12(48.00)	13 (52.00)		12 (46.15)	14 (53.85)		9 (36.00)	16 (64.00)		9 (36.00)	16 (64.00)	
**Member of a social group**													
No	33 (55.93)	16 (48.48)	17 (51.52)	0.908	17 (51.52)	16 (48.48)	0.318	13 (39.39)	20 (60.61)	0.415	18 (54.55)	15 (45.45)	0.351
Yes	26 (44.07)	13 (50.00)	13 (50.00)		10 (38.46)	16 (61.54)		13 (50.00)	13 (50.00)		11 (42.31)	15 (57.69)	
**Participated in DeWorm3 community sensitisation activities**													
No	30 (50.85)	17 (56.67)	13 (43.33)	0.240	19 (63.33)	11 (36.67)	**0.027** [c]	15 (50.00)	15 (50.00)	0.351	16 (53.33)	14 (46.67)	0.514
Yes	29 (49.15)	12 (41.38)	17 (58.62)		10 (34.48)	19 (65.52)		11 (37.93)	18 (62.07)		13 (44.83)	16 (55.17)	
**Familiar with community drug distributor**													
No	7 (11.86)	4 (57.14)	3 (42.86)	0.706 [b]	4 (57.14)	3 (42.86)	0.652	3 (42.86)	4 (57.14)	1.000 [b]	3 (42.86)	4 (57.14)	1.000 [b]
Yes	52 (88.14)	25 (48.08)	27 (51.92)		25 (48.08)	27 (51.92)		23 (44.23)	29 (55.77)		26 (50.00)	26 (50.00)	
**Perceived severity of the adverse event**													
Severe	34 (57.63)	16 (47.06)	18 (52.94)	0.708	19 (55.88)	15 (44.12)	0.228	17 (50.00)	17 (50.00)	0.284	16 (47.06)	18 (52.94)	0.708
Moderate/mild	25 (42.37)	13 (52.00)	12 (48.00)		10 (40.00)	15 (60.00)		9 (36.00)	16 (64.00)		13 (52.00)	12 (48.00)	

[a] Others–unmarried/widowed/separated; [b] Below grade 10; [c] Others–Christians/Muslims; [d] three or more generations living together or living with relatives; [e] Skilled–tailoring/carpentry/welding/mechanics; [f] Social group (community-based groups)–women's self-help groups/farmers' club/cooperative societies; [g] Fisher's exact test for factors with less than 5 expected cell counts, [h] Significant at p-value ≤0.05

Acronyms: SC/ST-Scheduled Caste/Scheduled Tribe, MBC/BC/FC - Most Backward Class/Backward Class/Forward Class

Individuals with strong ties to family and community are more likely to believe that those around them are participating in cMDA

Most of the participants believed that their neighbours and friends participated in cMDAs (85%), and that people in their community considered it important to control or stop the spread of STH (73%). Many participants thought that their neighbours and friends would expect them to participate in cMDAs (63%), and few of them felt that their participation will be influenced by their neighbours' and friends' participation (10%). These findings corroborated with results of the in-depth interviews, where most participants reported that everyone in their families, as well as people in their village, were taking the tablet and continued to take it because of its health benefits. They said,

> "*People do think of completely eradicating (STH). Everyone in the village thinks that these intestinal worms would not be generated again by eating this tablet. Those who eat this tablet will benefit from it.*" (Woman #1- perceived moderately severe AE, Cluster 38)

> "*I eat the tablet when it is given to me, similarly others also say…. Yes, neighbours ask… They ask, "Did you get the tablet and eat?" I say that I did take the tablet and eat.*" (Woman #3- perceived moderately severe AE, Cluster 38)

A high score for subjective norm indicates individuals perception that the people around them participate in cMDAs and would like to control or stop STH transmission. Overall, 51% of the participants showed a high score for subjective norm (≥median score of 18; maximum score– 20) for STH cMDA. A high subjective norm score was significantly associated with family type and prior participation in DeWorm3 community sensitisation activities (χ^2=7.50, $P<0.05$; and χ^2=4.91, $P<0.05$ respectively) (Table 1). Among those who belonged to extended families, 67% had high subjective norm scores compared to those who belonged to nuclear families

(31%). Among those who participated in DeWorm3 community sensitisation activities, 69% had high subjective norm scores compared to those who did not (37%).

Individuals engaged in skilled occupations had higher perceived behavioural control in participating in cMDAs

Behavioural control reflects individuals' self-efficacy to engage in cMDA. Most participants were confident that they would be able to access deworming tablets distributed during cMDA campaigns if they wanted to (90%), and they would participate in future STH cMDAs even if their neighbours and friends tell them not to participate (78%). Many of them (76%) reported that buying deworming tablets from private medical stores for their family members was prohibitively expensive. Most participants reported that they would participate in future STH cMDAs (85%) and would not wait for approval from any family member (61%). In-depth interviews confirmed that most individuals feel free to independently make decisions about their own treatment, while a smaller minority are influenced by family members,

> "*First time when they gave the tablet got diarrhoea… next time my family also said not to eat… I ate a tablet the second time… It is not that I will eat only when my family members tell me to eat. It is my choice.*" (Woman #1- perceived moderately severe AE, Cluster 38)

> "*I will eat. My son said, 'ma, you eat the tablet. On that day, something unfortunate happened.' Then I must listen to my eldest son. When he tells, I will think about what my son had said.*" (Woman #1- perceived moderately severe AE, Cluster 24)

Overall, 56 % of the participants had a higher perceived behavioural control (≥median score of 25; maximum score–27) to participate in cMDA. Perceived behavioural control was significantly associated with participants' occupation (χ^2=5.02, P<0.05) (Table 1). Those

engaged in skilled occupations (78%) such as tailoring, carpentry, welding, mechanics etc. had higher perceived behavioural control than those engaged in unskilled/semi-skilled occupation such as daily labourers, peons, and security guards (46%).

Intention to participate in future cMDAs was high among the disadvantaged socio-economic groups (scheduled caste/scheduled tribe) and extended families

A majority of participants (85%) said they would consume or let their child consume the tablet in the future, although one third (32%) believed that the tablet could cause an AE and were concerned about it for themselves and their family members. The in-depth interviews showed that the individuals' decision to participate was based on the perceived health benefit despite experiencing an AE because deworming relieved them of stomach pain, increased their appetite, and they felt more energetic to perform their work. They said,

> *"After eating the tablet I was able to move quickly from place to place, my body was active…. I do not have intestinal worms so I think I am healthy. Once in six months I eat tablet. My menstrual cycle is regular when I eat the tablet."* (Woman #1- perceived moderately severe AE, Cluster 23)

> *"It is good to eat the tablet, Sir. We feel well after eating the tablet, we do not have intestinal worms. Where I work, many ladies from this village and the neighbouring village come, they also say that their body feels well after eating the tablet."* (Woman #1- perceived moderately severe AE, Cluster 12)

Overall, 51% of the participants had a high intention to participate in future cMDAs (≥median score of 18; maximum score–20). A high level of intention to participate was significantly associated with caste and family type (χ^2=3.83, P=0.050 and χ^2=7.50, P<0.05 respectively)

(Table 1). Intention to participate was higher among those who belonged to SC/ST (62%) than those who belonged to other castes (36%) and among those belonging to extended families (67%) compared to nuclear families (31%).

Attitude and perceived behavioural control were significant predictors of intention to participate

The framework of TPB wherein attitude, subjective norm, and perceived behavioural control are hypothesised to have a relationship with intention to participate in cMDA was used to inform a path analysis (Figure 3). The path model showed a good fit for the data ($\chi 2$ =29.80, $P < 0.001$), accounting for 39.7% variance in intention to participate. Attitude and perceived behavioural control (composite scores) were significant predictors of future intention to participate in cMDA. The mean value of intention to participate increased by 0.28 for every unit change in attitude and by 0.33 for every unit change in perceived behavioural control. Subjective norms did not emerge as a significant predictor of intention to participate. The analysis also showed a significantly strong relationship between the independent variables of attitude, subjective norm and perceived behavioural control. An effect size (f^2) of 0.35, significance level (α) of 0.05, total sample size (n) of 59, and number of predictors (X) as 3 were used as parameters to compute the power of a statistical test for the path analysis, which resulted in an adequate statistical power ($1-\beta$) of 0.97.

Fig 3: Path model - the relationship between attitude, subjective norm, perceived behavioural control with intention to participate.

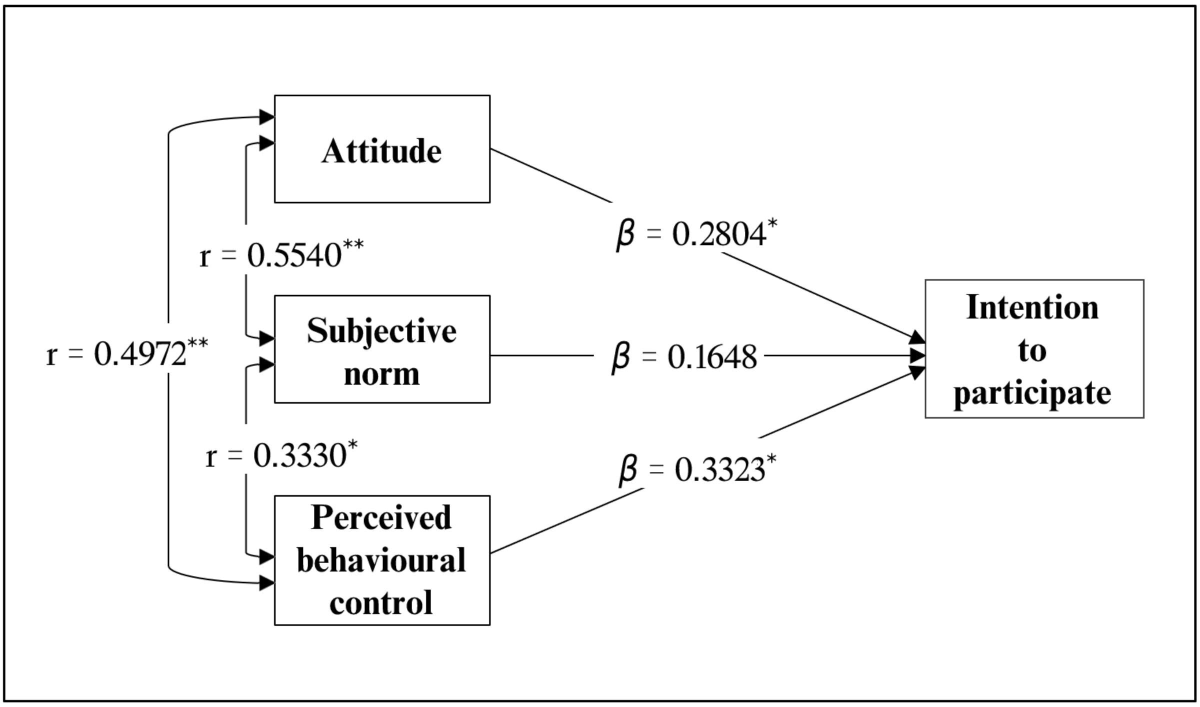

Note: All parameter values are standardised; *p<.05, **p<.001; r–correlation coefficients; β–regression coefficients, the outcome variable increases β times for every unit increase in a predictor variable.

Discussion

This paper examines the perspectives of those who reported an AE after taking albendazole during cMDA for STH and investigates the behavioural factors that influence their intention to participate in future rounds of deworming. In this study, as also observed in studies in multiple other countries including India, more women than men and children reported experiencing an AEs during MDA (33). However, in this study, participation in cMDA after an AE was high, with only 12% refusing to participate in the next cMDA and only 4% refusing to participate in all subsequent cMDA rounds. These rates are similar to other studies that have evaluated the impact of AEs on participation in MDA programs; studies in Ethiopia and malaria in Gambia have reported refusal to participate in MDA ranging between 7–33% as a result of individuals having experienced an AE (34-36).

Intention to participate in future cMDA was also significantly higher among those who belonged to SC/ST (a caste group officially designated the most disadvantaged socio-economic groups in India), and extended families, possibly because of the high perceived risk of STH infection and family support to participate in cMDAs, as shown in the in-depth interviews. Attitude and perceived behavioural control were significant predictors of intention to participate in future STH cMDA. Despite experiencing an AE, most participants believed that the tablet was safe and effective probably because they considered deworming to be good for their health (37). A positive attitude (≥ median composite score) towards cMDA was significantly higher among the most disadvantaged households who belonged to SC/ST. Another earlier study by our team demonstrated that a higher proportion of CDDs who belong to SC/ST households have a positive attitude towards cMDA (38). The likelihood of perceived risk to STH infections may be higher among SC/ST households as they have poor water and sanitation facilities and less access to healthcare (39). Overall, half of the participants believed that STH is a minor infection (49%), and deworming was not necessary for those who use toilets (53%). Therefore, messages on STH-related morbidities and the importance of high treatment coverage for STH elimination need to be incorporated into community sensitisation activities. Individuals engaged in skilled occupations were found to have higher perceived behavioural control in participating in cMDA, possibly due to increased agency as a result of higher levels of freedom of movement, decision-making capacity, and income. A Sri Lankan study on LF MDA program also showed that participation was higher among skilled workers in the middle-income group as compared to those in lower- and upper-income groups (40).

Subjective norm values, or the individual's perceived social expectations, were significantly higher among individuals with strong ties to family and community. These individuals are more likely to believe that those around them are participating in cMDA. This may have been shaped

by observing community members' responses and expectations during community sensitisation activities and the intrahousehold discussion about community norms concerning cMDAs. However, path analysis showed no significant relationship between subjective norm and intention to participate. Analysis of two studies on voting and energy consumption that examined the interactions between attitude, subjective norm, and perceived behavioural control on the prediction of intention showed that higher perceived behavioural control tends to strengthen the attitude and weakens subjective norm in the prediction of intention (41). This study also showed a significantly higher correlation of perceived behavioural control with attitude than with subjective norms.

Participation in subsequent cMDAs after an AE was high in our study. However, about two-thirds of the participants in our study were concerned about the occurrence of an AE in the future. The in-depth interviews also revealed that when the perceived AE is perceived as being severe, it could even lead to household-level refusal or refusal to treat their children. Such non-compliers may serve as a source of STH infection, as observed in an LF-MDA study in Haiti (42). Community members in an LF-MDA study in India suggested that CDDs should be given training and drugs to manage AEs as concerns about AEs persisted even though AEs reduced over the course of repeated MDAs (15,42,43). Our qualitative research also demonstrates that CDDs were often the first point of contact for community members when they experienced the AE, therefore, MDA programs will benefit from providing counselling to community members to reduce fear, particularly among those experiencing AEs. Adopting a door-step treatment delivery strategy provides an opportunity to counsel those who fear an AE. Messages on the management of common side-effects may be helpful as about 10% of the non-compilers in an LF-MDA study from Odisha in India reported that they would have complied if they knew how to manage the side-effects (44,45). Incorporating messages in community sensitization

activities on AEs, that the drug is safe and the side-effects are short-term as well as about access to care in case of an AE, and management of AE by qualified health personnel in the community may be critical to optimize successful implementation of MDA programs with high coverage (44).

Strengths and Limitations

This study had several notable strengths. The surveys and interviews were conducted within the context of a highly rigorous randomized trial in which detailed information allowed the identification of all individuals with reported AEs. Participation rates were high and the repeated cMDA rounds within the parent trial allowed for an assessment of behaviour over time. However, this analysis also had limitations. AE surveillance was conducted passively, so it is possible that some AEs of mild or even moderate severity may not have been reported. Measuring only the intention to participate in future cMDAs instead of actual behaviour was not possible in this study because the trial included only six rounds of cMDA. Additionally, there could be recall bias as we carried out this research six months after completion of all six rounds of cMDA due to delays driven by the COVID-19 pandemic.

Conclusion

This study demonstrates that positive attitude and perceived behavioural control have a significant association with the intention to participate in a future round of cMDA, even after experiencing an AE. Caste (SC/ST households), family type (extended families), occupation (engaged in skilled occupation), and participation in community sensitisation activities were factors significantly associated with various TBP constructs. Messages on AE and its management need to be incorporated in community mobilization activities and specifically aimed at castes other than SC/ST and nuclear families. CDDs need to be oriented about AEs

during cMDAs as they are important stakeholders in mitigating the fear of AEs. In addition, research using the TPB framework with larger sample sizes is required to better inform MDA programs about behavioural factors influencing intention to participate after an AE.

Acknowledgements

The authors wish to thank all of the study participants, communities and community leaders, national NTD program staff and other local, regional and national partners who have participated or supported the DeWorm3 study. We also would like to acknowledge the work of all members of the DeWorm3 study teams and affiliated institutions. We give special thanks to Isaac Arputharangam, and Prasanna Yesuratinam for their support in data collection, and the field managers, Rajeshkumar Rajendiran and Chinnaduraipandi Paulsamy, for providing logistical support in the field. We thank Naveenkumar Sekar for creating the data entry form of the questionnaire and Uma Maheshwari for her support in transcribing qualitative audio files.

References

1. Lai YS, Biedermann P, Shrestha A, Chammartin F, a Porta N, Montresor A, Mistry NF, Utzinger J, Vounatsou P. Risk profiling of soil-transmitted helminth infection and estimated number of infected people in South Asia: A systematic review and Bayesian geostatistical Analysis. PLoS neglected tropical diseases. 2019 Aug 9;13(8):e0007580. https://doi.org/10.1371/journal.pntd.0007580

2. World Health Organization. 2030 targets for soil-transmitted helminthiases control programmes. Geneva: World Health Organization, Geneva; 2019. [cited 9 May 2022].

Available from: https://apps.who.int/iris/bitstream/handle/10665/330611/9789240000315-eng.pdf

3. Anderson RM, Truscott JE, Pullan RL, Brooker SJ, Hollingsworth TD. How effective is school-based deworming for the community-wide control of soil-transmitted helminths?. PLoS neglected tropical diseases. 2013 Feb 28;7(2):e2027. https://doi.org/10.1371/journal.pntd.0002027

4. Truscott JE, Turner HC, Anderson RM. What impact will the achievement of the current World Health Organisation targets for anthelmintic treatment coverage in children have on the intensity of soil transmitted helminth infections?. Parasites & vectors. 2015 Dec;8(1):1-2. https://doi.org/10.1186/s13071-015-1135-4

5. Brooker SJ, Nikolay B, Balabanova D, Pullan RL. Global feasibility assessment of interrupting the transmission of soil-transmitted helminths: a statistical modelling study. The Lancet Infectious Diseases. 2015 Aug;15(8):941–50. https://doi.org/10.1016/S1473-3099(15)70042-3

6. Pullan RL, Halliday KE, Oswald WE, Mcharo C, Beaumont E, Kepha S, Witek-McManus S, Gichuki PM, Allen E, Drake T, Pitt C. Effects, equity, and cost of school-based and community-wide treatment strategies for soil-transmitted helminths in Kenya: a cluster-randomised controlled trial. The Lancet. 2019 May 18;393(10185):2039-50. https://doi.org/10.1016/S0140-6736(18)32591-1

7. DeWorm3 Trials Team. Baseline patterns of infection in regions of Benin, Malawi and India seeking to interrupt transmission of soil transmitted helminths (STH) in the DeWorm3 trial. PLoS neglected tropical diseases. 2020 Nov 2;14(11):e0008771. https://doi.org/10.1371/journal.pntd.0008771

8. Ásbjörnsdóttir KH, Ajjampur SS, Anderson RM, Bailey R, Gardiner I, Halliday KE, Ibikounle M, Kalua K, Kang G, Littlewood DT, Luty AJ. Assessing the feasibility of interrupting the transmission of soil-transmitted helminths through mass drug administration: The DeWorm3 cluster randomized trial protocol. PLoS neglected tropical diseases. 2018 Jan 18;12(1):e0006166. https://doi.org/10.1371/journal.pntd.0006166

9. Means AR, Ajjampur SS, Bailey R, Galactionova K, Gwayi-Chore MC, Halliday K, Ibikounle M, Juvekar S, Kalua K, Kang G, Lele P. Evaluating the sustainability, scalability, and replicability of an STH transmission interruption intervention: The DeWorm3 implementation science protocol. PLoS neglected tropical diseases. 2018 Jan 18;12(1):e0005988. https://doi.org/10.1371/journal.pntd.0005988

10. Banerjee S, Bandyopadhyay K, Khan MF, Akkilagunta S, Selvaraj K, Tripathy JP, Solanki R, Kushwaha AS, Deshmukh P. Coverage of mass drug administration for elimination of lymphatic filariasis in urban Nagpur, Central India: A mixed method study. Journal of Family Medicine and Primary Care. 2019 Sep;8(9):3009. doi:10.4103/jfmpc.jfmpc_503_19

11. Ramaiah KD, Das PK, Appavoo NC, Ramu K, Augustin DJ, Vijay Kumar KN, Chandrakala AV. A programme to eliminate lymphatic filariasis in Tamil Nadu state, India: compliance with annual single- dose DEC mass treatment and some related operational aspects. Tropical Medicine & International Health. 2000 Nov;5(12):842-7.

12. Babu BV, Kar SK. Coverage, compliance and some operational issues of mass drug administration during the programme to eliminate lymphatic filariasis in Orissa, India. Tropical Medicine & International Health. 2004 Jun;9(6):702-9.

13. Karmakar PR, Mitra K, Chatterjee A, Jana PK, Bhattacharya S, Lahiri SK. A study on coverage, compliance and awareness about mass drug administration for elimination of lymphatic filariasis in a district of West Bengal, India. Journal of vector borne diseases. 2011 Jun 1;48(2):101.

14. Hussain MA, Sitha AK, Swain S, Kadam S, Pati S. Mass drug administration for lymphatic filariasis elimination in a coastal state of India: a study on barriers to coverage and compliance. Infectious diseases of poverty. 2014 Sep;3(1):1-8.

15. Lahariya C, Mishra A. Strengthening of mass drug administration implementation is required to eliminate lymphatic filariasis from India: an evaluation study. The Journal of Vector Borne Diseases. 2008 Dec 1;45(4):313-20.

16. Government of India. National Deworming Day: Operational Guidelines [Internet]. Ministry of Health & Family Welfare; 2015 [cited 2021 Jan 22]. Available from: https://www.nhp.gov.in/national-deworming-day_pg

17. Government of India M of H& FW. Accelerated Plan for Eliminate of Lymphatic Filariasis [Internet]. [cited 2021 May 28]. Available from: https://nvbdcp.gov.in/WriteReadData/l892s/1031567531528881007.pdf

18. Aswathy S, Beteena K, Leelamoni K. Mass drug administration against filariasis in India: perceptions and practices in a rural community in Kerala. Annals of Tropical Medicine & Parasitology. 2009 Oct;103(7):617–24. https://doi.org/10.1179/000349809X12459740922255

19. Roy RN, Sarkar AP, Misra R, Chakroborty A, Mondal TK, Bag K. Coverage and Awareness of and Compliance with Mass Drug Administration for Elimination of Lymphatic Filariasis in Burdwan District, West Bengal, India. Journal of Health,

Population and Nutrition. 2013 Sep 9;31(2):171–7. doi: 10.3329/jhpn.v31i2.16380 PMID: 23930334

20. Ajzen I. The theory of planned behavior. Organizational behavior and human decision processes. 1991 Dec;50(2):179–211.

21. Jadgal KM, Zareban I, Rakhshani F, Shahrakipour M, Sepehrvand B, Alizadeh Sivaki H. The effect of health education according to the theory of planned behavior on malaria preventive behavior in rural men of Chabahar. Journal of Research and Health. 2012 Oct 10;2(2):237-46.

22. Putra MM, Sari NP. Model Theory of Planned Behavior to Improve Adherence to Treatment and the Quality of Life in Tuberculosis Patients. Jurnal Ners. 2020 Oct;15(2):167-72. http://dx.doi.org/10.20473/jn.v15i1.17958

23. Gibson LP, Magnan RE, Kramer EB, Bryan AD. Theory of Planned Behavior Analysis of Social Distancing During the COVID-19 Pandemic: Focusing on the Intention–Behavior Gap. Annals of Behavioral Medicine. 2021 Jul 22;55(8):805–12. doi: 10.1093/abm/kaab041 PMID: 34228112

24. Yu Y, Lau JTF, Lau MMC. Levels and factors of social and physical distancing based on the Theory of Planned Behavior during the COVID-19 pandemic among Chinese adults. Translational Behavioral Medicine. 2021 May 25;11(5):1179–86. doi: 10.1093/tbm/ibaa146 PMID: 33598679

25. Shmueli L. Predicting intention to receive COVID-19 vaccine among the general population using the health belief model and the theory of planned behavior model. BMC Public Health. 2021 Dec;21(1):1-3.

26. Wolff K. COVID-19 vaccination intentions: the theory of planned behavior, optimistic bias, and anticipated regret. Frontiers in Psychology. 2021:2404. https://doi.org/10.3389/fpsyg.2021.648289

27. Irfan M, Akhtar N, Ahmad M, Shahzad F, Elavarasan RM, Wu H, Yang C. Assessing public willingness to wear face masks during the COVID-19 pandemic: fresh insights from the theory of planned behavior. International Journal of Environmental Research and Public Health. 2021 Jan;18(9):4577. https://doi.org/10.3390/ijerph18094577

28. Thaker J, Ganchoudhuri S. The Role of Attitudes, Norms, and Efficacy on Shifting COVID-19 Vaccine Intentions: A Longitudinal Study of COVID-19 Vaccination Intentions in New Zealand. Vaccines. 2021 Oct 4;9(10):1132. https://doi.org/10.3390/vaccines9101132

29. Ajjampur SS, Kaliappan SP, Halliday KE, Palanisamy G, Farzana J, Manuel M, Abraham D, Laxmanan S, Aruldas K, Rose A, Kennedy DS. Epidemiology of soil transmitted helminths and risk analysis of hookworm infections in the community: Results from the DeWorm3 Trial in southern India. PLoS neglected tropical diseases. 2021 Apr 30;15(4):e0009338. https://doi.org/10.1371/journal.pntd.0009338

30. Harpe SE. How to analyze Likert and other rating scale data. Currents in Pharmacy Teaching and Learning. 2015 Nov;7(6):836–50.

31. Tavakol M, Dennick R. Making sense of Cronbach's alpha. International journal of medical education. 2011;2:53. doi: 10.5116/ijme.4dfb.8dfd PMID: 28029643

32. Streiner DL. Finding our way: an introduction to path analysis. The Canadian Journal of Psychiatry. 2005 Feb;50(2):115-22.

33. Weil GJ, Bogus J, Christian M, Dubray C, Djuardi Y, Fischer PU, Goss CW, Hardy M, Jambulingam P, King CL, Kuttiat VS. The safety of double-and triple-drug community mass drug administration for lymphatic filariasis: A multicenter, open-label, cluster-randomized study. PLoS medicine. 2019 Jun 24;16(6):e1002839. https://doi.org/10.1371/journal.pmed.1002839

34. Offei M, Anto F. Compliance to mass drug administration programme for lymphatic filariasis elimination by community members and volunteers in the Ahanta West District of Ghana. Journal of Bacteriology & Parasitology. 2014 Jan 1;5(1):1.

35. Astale T, Sata E, Zerihun M, Nute AW, Stewart AE, Gessese D, Ayenew G, Melak B, Chanyalew M, Tadesse Z, Callahan EK. Population-based coverage survey results following the mass drug administration of azithromycin for the treatment of trachoma in Amhara, Ethiopia. PLoS neglected tropical diseases. 2018 Feb 16;12(2):e0006270. https://doi.org/10.1371/journal.pntd.0006270

36. De Martin S, Von Seidlein L, Deen JL, Pinder M, Walraven G, Greenwood B. Community perceptions of a mass administration of an antimalarial drug combination in The Gambia. Tropical medicine & international health. 2001 Jun;6(6):442-8.

37. Aruldas K, Means AR, Titus A, Jacob Y, Rajendiran R, Johnson J, Emmanuel-Fabula M, Puthupalayam Kaliappan S, Kamlakar Juvekar S, Kang G, L. Walson J. Gender differences in the perceived need for community-wide deworming: Formative qualitative research from the DeWorm3 study, India. PLoS neglected tropical diseases. 2020 Nov 25;14(11):e0008829. https://doi.org/10.1371/journal.pntd.0008829

38. Aruldas K, Kaliappan SP, Israel GJ, Palanisamy G, Johnson J, Titus A, Walson JL, Means AR, Ajjampur SS. Community Drug Distributor Knowledge, Attitudes, and

Motivation Surrounding Mass Drug Administration for Soil-Transmitted Helminths in India. Frontiers in public health. 2021:1827. https://doi.org/10.3389/fpubh.2021.714606

39. Strunz EC, Addiss DG, Stocks ME, Ogden S, Utzinger J, Freeman MC. Water, sanitation, hygiene, and soil-transmitted helminth infection: a systematic review and meta-analysis. PLoS medicine. 2014 Mar 25;11(3):e1001620. https://doi.org/10.1371/journal.pmed.1001620

40. Gunawardena S, Ismail M, Bradley M, Karunaweera N. Factors influencing drug compliance in the mass drug administration programme against filariasis in the Western province of Sri Lanka. Transactions of the Royal Society of Tropical Medicine and Hygiene. 2007 May 1;101(5):445-53.

41. La Barbera F, Ajzen I. Control interactions in the theory of planned behavior: Rethinking the role of subjective norm. Europe's Journal of Psychology. 2020 Aug;16(3):401. doi: 10.5964/ejop.v16i3.2056 PMID: 33680190

42. Boyd A, Won KY, McClintock SK, Donovan CV, Laney SJ, Williams SA, Pilotte N, Streit TG, Beau de Rochars MV, Lammie PJ. A community-based study of factors associated with continuing transmission of lymphatic filariasis in Leogane, Haiti. PLoS neglected tropical diseases. 2010 Mar 23;4(3):e640. https://doi.org/10.1371/journal.pntd.0000640

43. Krentel A, Fischer PU, Weil GJ. A review of factors that influence individual compliance with mass drug administration for elimination of lymphatic filariasis. PLoS neglected tropical diseases. 2013 Nov 21;7(11):e2447. https://doi.org/10.1371/journal.pntd.0002447

44. Silumbwe A, Zulu JM, Halwindi H, Jacobs C, Zgambo J, Dambe R, Chola M, Chongwe G, Michelo C. A systematic review of factors that shape implementation of mass drug administration for lymphatic filariasis in sub-Saharan Africa. BMC public health. 2017 Dec;17(1):1-5. DOI 10.1186/s12889-017-4414-5.

45. Cantey PT, Rout J, Rao G, Williamson J, Fox LM. Increasing compliance with mass drug administration programs for lymphatic filariasis in India through education and lymphedema management programs. PLoS neglected tropical diseases. 2010 Jun 29;4(6):e728. https://doi.org/10.1371/journal.pntd.0000728

Supporting Information (S1 file)

Click here to access/download;Figure;Fig 1.tiff

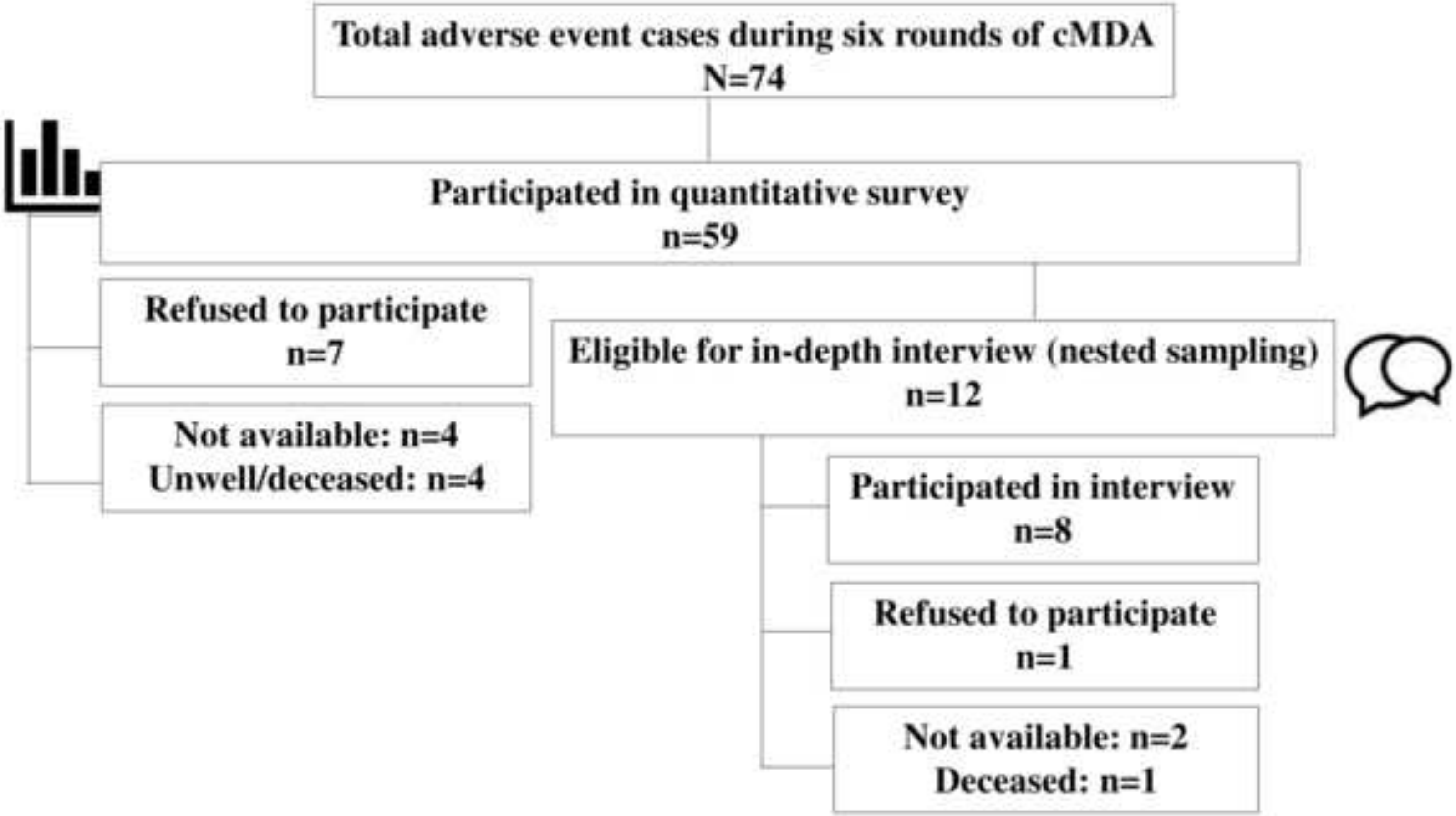

Figure

Click here to access/download;Figure;Fig 2.tiff

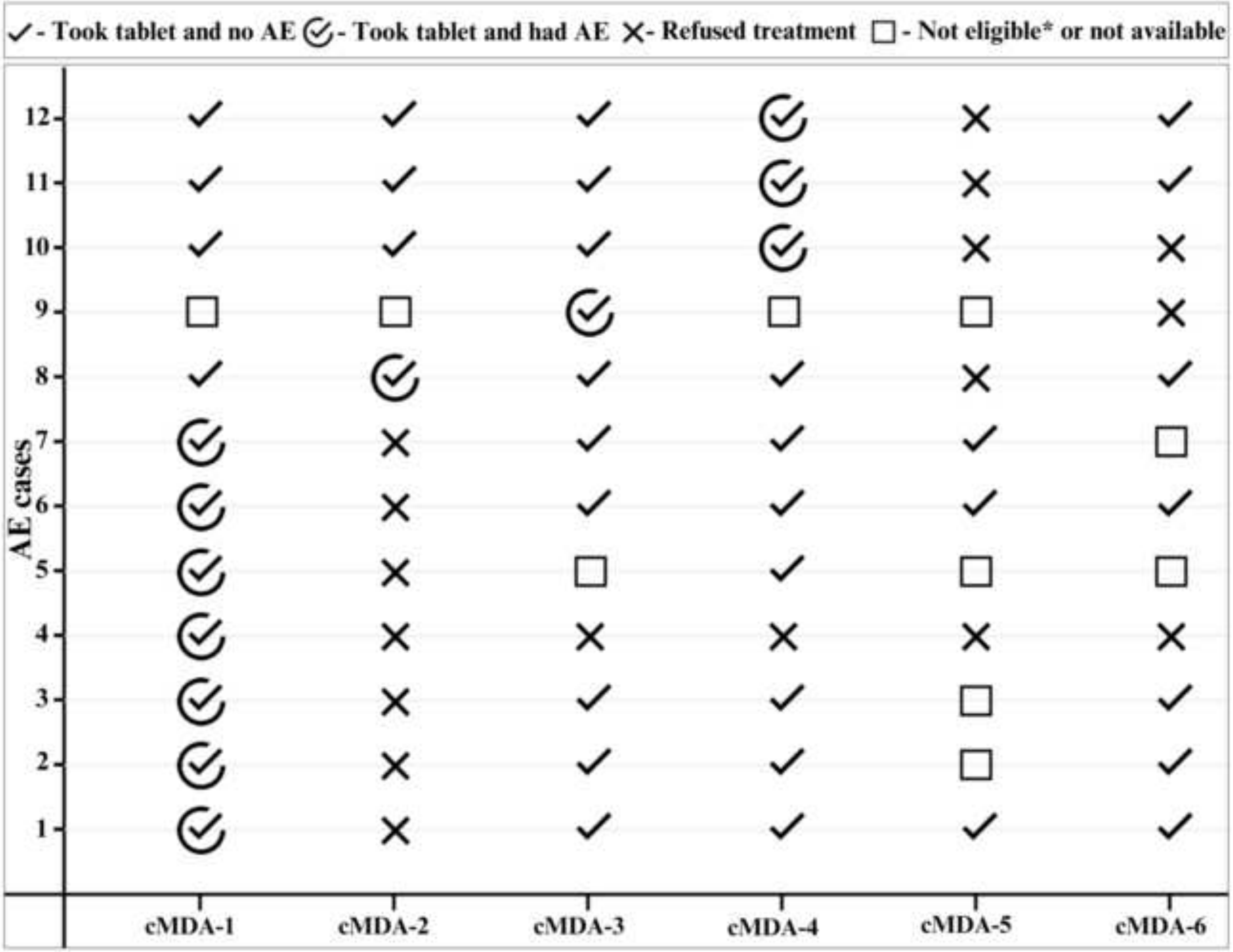

Click here to access/download;Figure;Fig 3.tiff

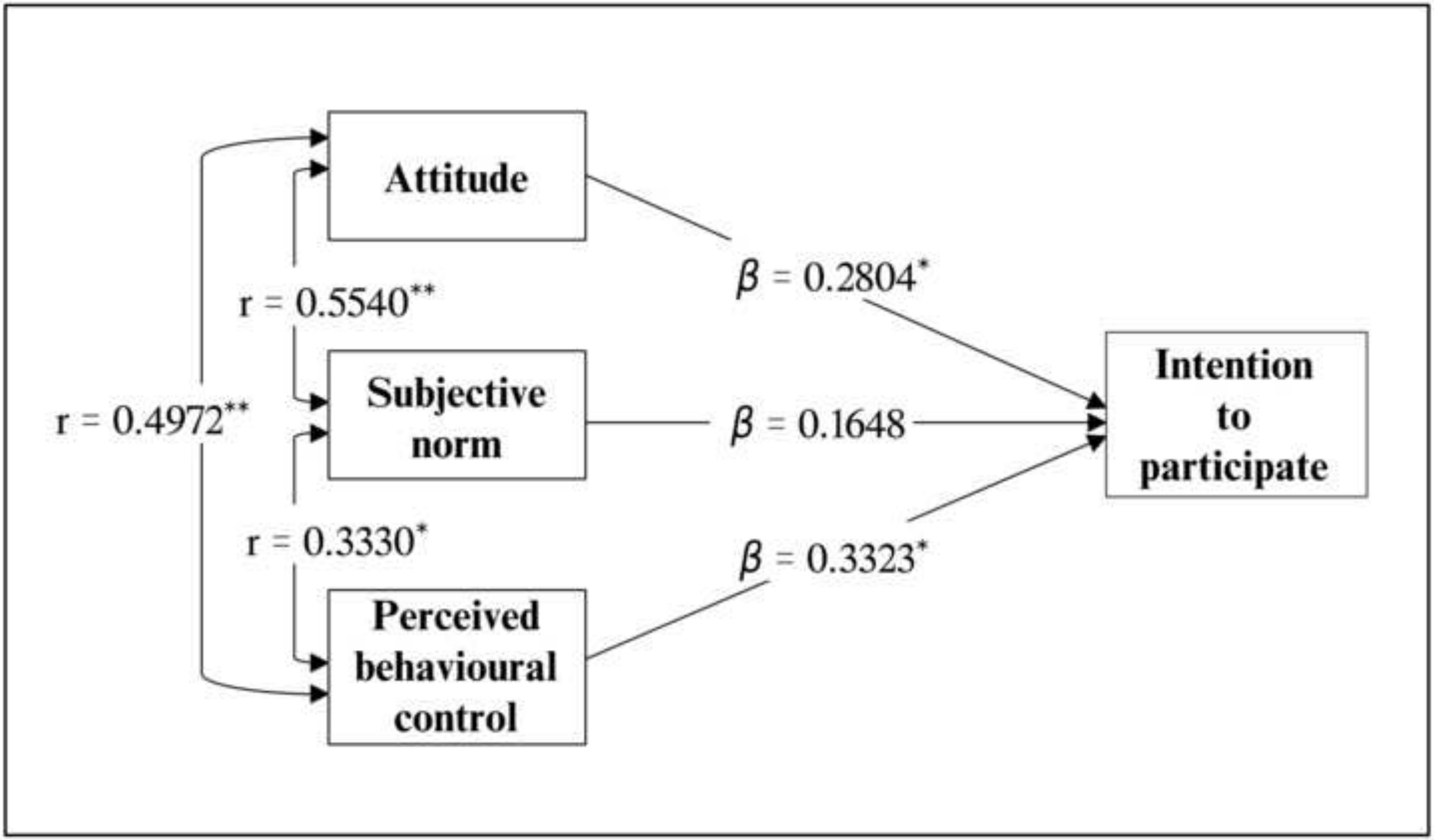

Table Click here to access/download;Table;Table 1.docx

Table 1. Factors associated with attitude, subjective norm, perceived behavioural control, and intention to participate in STH cMDA

Factors	Total	Attitude			Subjective norm			Perceived behavioural control			Intention to participate		
	n=59	Low n=29	High n=30	Chi-Square test	Low n=29	High n=30	Chi-Square test	Low n=26	High n=33	Chi-Square test	Low n=29	High n=30	Chi-Square test
	n (%)	n (%)	n (%)	p-value	n (%)	n (%)	p-value	n (%)	n (%)	p-value	n (%)	n (%)	p-value
**Sex**													
Male	16 (27.12)	11 (68.75)	5 (31.25)	**0.066**	8 (50.00)	8 (50.00)	0.937	9 (56.25)	7 (43.75)	0.250	10 (62.50)	6 (37.50)	0.211
Female	43 (72.88)	18 (41.86)	25 (58.14)		21 (48.84)	22 (51.16)		17 (39.53)	26 (60.47)		19 (44.19)	24 (55.81)	
**Age**													
Below median	28 (47.46)	12 (42.86)	16 (57.14)	0.358	16 (57.14)	12 (42.86)	0.243	13 (46.43)	15 (53.57)	0.728	12 (42.86)	16 (57.14)	0.358
Above median	31 (52.54)	17 (54.84)	14 (45.16)		13 (41.94)	18 (58.06)		13 (41.94)	18 (58.06)		17 (54.84)	14 (45.16)	
**Marital status**													
Married	44 (74.58)	19 (43.18)	25 (56.82)	0.116	19 (43.18)	25 (56.82)	0.116	18 (40.91)	26 (59.09)	0.403	20 (45.45)	24 (54.55)	0.33
Others [a]	15 (25.42)	10 (66.67)	5 (33.33)		10 (66.67)	5 (33.33)		8 (53.33)	7 (46.67)		9 (60.00)	6 (40.00)	
**Education**													
Below secondary	39 (66.10)	22 (56.41)	17 (43.59)	0.119	19 (48.72)	20 (51.28)	0.926	19 (48.72)	20 (51.28)	0.315	21 (53.85)	18 (46.15)	0.314
Secondary and above	20 (33.90)	7 (35.00)	13 (65.00)		10 (50.00)	10 (50.00)		7 (35.00)	13 (65.00)		8 (40.00)	12 (60.00)	
**Highest level of household education**													
Below secondary	12 (20.34)	6 (50.00)	6 (50.00)	0.948	7 (58.33)	5 (41.67)	0.476	5 (41.67)	7 (58.33)	1.000 [b]	3 (25.00)	9 (75.00)	**0.061**
Secondary and above	47 (79.66)	23 (48.94)	24 (51.06)		22 (46.81)	25 (53.19)		21 (44.68)	26 (55.32)		26 (55.32)	21 (44.68)	
**Religion**													
Hindu	55 (93.22)	27 (49.09)	28 (50.91)	1.000 [b]	27 (49.09)	28 (50.91)	1.000 [b]	24 (43.64)	31 (56.36)	0.805	28 (50.91)	27 (49.06)	0.612 [b]
Others	4 (6.78)	2 (50.00)	2 (50.00)		2 (50.00)	2 (50.00)		2 (50.00)	2 (50.00)		1 (25.00)	3 (75.00)	
**Caste**													
SC/ST	34 (57.63)	13 (38.24)	21 (61.76)	**0.050** [c]	15 (44.12)	19 (55.88)	0.367	12 (35.29)	22 (64.71)	0.113	13 (38.24)	21 (61.76)	**0.050** [c]
Others (MBC/BC/FC)	25 (42.37)	16 (64.00)	9 (36.00)		14 (56.00)	11 (44.00)		14 (56.00)	11 (44.00)		16 (64.00)	9 (36.00)	
**Family type**													
Nuclear family	26 (44.07)	14 (53.85)	12 (46.15)	0.522	18 (69.23)	8 (30.77)	**0.006** [c]	13 (50.00)	13 (50.00)	0.415	18 (69.23)	8 (30.77)	**0.006** [c]
Extended family	33 (55.93)	15 (45.45)	18 (54.55)		11 (33.33)	22 (66.67)		13 (39.39)	20 (60.61)		11 (33.33)	22 (66.67)	

**Occupation**													
Unskilled/Semi-skilled	37 (67.27)	21 (51.22)	20 (48.78)	0.504	20 (48.78)	21 (51.22)	0.931	22 (53.66)	19 (46.34)	**0.025** [c]	19 (46.34)	22 (53.66)	0.391
Skilled	18 (32.73)	8 (44.44)	10 (55.56)		9 (50.00)	9 (50.00)		4 (22.22)	14 (77.78)		10 (55.56)	8 (44.44)	
**Highest level of household occupation**													
Unskilled/Semi-skilled	48 (87.27)	23 (44.23)	29 (55.77)	0.101 [b]	25 (48.08)	27 (51.92)	0.706 [b]	24 (46.15)	28 (53.85)	0.449 [b]	25 (48.08)	27 (51.92)	0.696 [b]
Skilled	7 (12.73)	6 (85.71)	1 (14.29)		4 (57.14)	3 (42.86)		2 (28.57)	5 (71.43)		4 (57.14)	3 (42.86)	
**Household toilet**													
No	34 (57.63)	17 (50.00)	17 (50.00)	0.879	17 (51.52)	16 (48.48)	0.683	17 (50.00)	17 (50.00)	0.284	20 (58.82)	14 (41.18)	**0.083**
Yes	25 (42.37)	12(48.00)	13 (52.00)		12 (46.15)	14 (53.85)		9 (36.00)	16 (64.00)		9 (36.00)	16 (64.00)	
**Member of a social group**													
No	33 (55.93)	16 (48.48)	17 (51.52)	0.908	17 (51.52)	16 (48.48)	0.318	13 (39.39)	20 (60.61)	0.415	18 (54.55)	15 (45.45)	0.351
Yes	26 (44.07)	13 (50.00)	13 (50.00)		10 (38.46)	16 (61.54)		13 (50.00)	13 (50.00)		11 (42.31)	15 (57.69)	
**Participated in DeWorm3 community sensitisation activities**													
No	30 (50.85)	17 (56.67)	13 (43.33)	0.240	19 (63.33)	11 (36.67)	**0.027** [c]	15 (50.00)	15 (50.00)	0.351	16 (53.33)	14 (46.67)	0.514
Yes	29 (49.15)	12 (41.38)	17 (58.62)		10 (34.48)	19 (65.52)		11 (37.93)	18 (62.07)		13 (44.83)	16 (55.17)	
**Familiar with community drug distributor**													
No	7 (11.86)	4 (57.14)	3 (42.86)	0.706 [b]	4 (57.14)	3 (42.86)	0.652	3 (42.86)	4 (57.14)	1.000 [b]	3 (42.86)	4 (57.14)	1.000 [b]
Yes	52 (88.14)	25 (48.08)	27 (51.92)		25 (48.08)	27 (51.92)		23 (44.23)	29 (55.77)		26 (50.00)	26 (50.00)	
**Perceived severity of the adverse event**													
Severe	34 (57.63)	16 (47.06)	18 (52.94)	0.708	19 (55.88)	15 (44.12)	0.228	17 (50.00)	17 (50.00)	0.284	16 (47.06)	18 (52.94)	0.708
Moderate/mild	25 (42.37)	13 (52.00)	12 (48.00)		10 (40.00)	15 (60.00)		9 (36.00)	16 (64.00)		13 (52.00)	12 (48.00)	

[a] Others–unmarried/widowed/separated; [b] Below grade 10; [c] Others–Christians/Muslims; [d] three or more generations living together or living with relatives; [e] Skilled–tailoring/carpentry/welding/mechanics; [f] Social group (community-based groups)–women's self-help groups/farmers' club/cooperative societies; [g] Fisher's exact test for factors with less than 5 expected cell counts, [h] Significant at p-value ≤0.05
Acronyms: SC/ST-Scheduled Caste/Scheduled Tribe, MBC/BC/FC - Most Backward Class/Backward Class/Forward Class

Click here to access/download
Supporting Information
S1 file .docx

Click here to access/download
Supporting Information
S2 file .doc
