# Supplementary material for: Impact of adverse events during community-wide mass drug administration for soil-transmitted helminths on subsequent participation–a Theory of Planned Behaviour analysis
Source: PLoS Negl Trop Dis. 2023 Mar 14;17(3):e0011148. doi: 10.1371/journal.pntd.0011148 (PMC10013912; doi:10.1371/journal.pntd.0011148)
Supplement: Attachment — Submitted filename: Rsponse to the reviewers.docx [file pntd.0011148.s004.docx]

January 27, 2023

Georgios Pappas and Eva Clark

Editors

PLOS Neglected Tropical Diseases (NTDs)

Re: PLOS Neglected Tropical Diseases submission (PNTD-D-22-01128)

Dear PLOS NTDs Editors,

We sincerely thank you and the reviewers for your comments on our manuscript titled: "Impact of adverse events during community-wide mass drug administration for soil-transmitted helminths on subsequent participation – a Theory of Planned Behaviour analysis." We have modified the manuscript in response to the comments and feedback. Our point-by-point responses are given below.

RESPONSE TO REVIEWERS

ABSTRACT and INTRODUCTION

Reviewer #2

The required corrections are written in detail in the article file.

Comments from the manuscript:

1) Why was this theory used?

**The Theory of Planned Behaviour (TPB) was used in this study because it predicts an individual's intention to modify or adopt a behaviour and it is clarified in page 5 line# 101-102 to state, “The Theory of Planned Behaviour (TPB) *predicts an individual's intention to modify or adopt a behaviour*.” This theory has been used in other infectious diseases like malaria, tuberculosis, COVID-19 (described on page 5 line# 113-118.**

2) The methodology of the study should be explained in more detail

**As suggested, the ‘methods’ of the abstract is revised to provide more information and ‘conclusions’ revised to include intention to participate. The key words are revised to indicate the study. The changes are as follows:**

**Page 2, line# 31-34: “This study was conducted using a multi-method quantitative and qualitative approach among 74 participants *who experienced an AE during STH cMDA conducted as part of the ongoing Deworm3 trial* and the 12 participants who subsequently refused cMDA treatment.”**

**Page 2, line# 50-52: “*Intention to participate in future STH cMDAs was associated with caste (SC/ST) and family type (extended families)*.”**

3) Comments in introduction - The place this theory used in other studies

**TPB was used in studies from Iran, Indonesia, USA, China, Pakistan, and Israel. The details with references are included on page 5, line# 112-118). “*Very few studies have used the TPB to assess participation or uptake of interventions for infectious diseases. These include assessment of interventions such as sleeping under an insecticide-treated mosquito net for malaria prevention in Iran and adherence to tuberculosis treatment in Indonesia. However, several studies have used the TPB to evaluate intentions to adopt COVID-19 related behaviour changes as preventive measures during the pandemic, such as maintaining physical distancing, wearing a mask, and vaccination uptake in a range of countries including the USA, China, Pakistan, and Israel*.”**

4) The place this theory used in this study

**Our study was carried out in the state of Tamil Nadu in India (Page 5, line # 119) as “*This multi-method study in the state of Tamil Nadu, India*, …”**

METHODS

Reviewer #1

The objectives of the study are clearly articulated in the paper with a testable hypothesis stated. The study design is appropriate and addressed the stated objectives as well. The study population is well-described and appropriate for the hypothesis being tested. As stated, though the statistical analysis was difficult to understand in certain portions the due to obvious limitations of one’s knowledge and understanding of statistics, the conclusions and recommendations are well aligned with the study results and therefore outcomes. I do not have any ethical or regulatory concerns regarding this study.

**Thank you very much for your kind feedback.**

Reviewer #2

The required corrections are written in detail in the article file.

Comments from the manuscript:

1) criteria for entering and exiting the study

**All cMDA participants who reported an adverse event after taking albendazole were eligible to be included in the study and there were no exclusion criteria (page 7, line # 152-154) “*The medical teams recorded 75 AEs among 74 participants (one participant reported AEs in two rounds of cMDA) during the six rounds of cMDA*.”**

2) Inclusion criteria for participating in STH cMDA

**All in the age of 1-99 years were eligible to participate in STH cMDA except for women in their first trimester of pregnancy, those with history of adverse reaction to benzimidazoles, and acutely ill at the time of MDA (page 6, line # 133-135) “*During the cMDA in the intervention clusters, the community drug distributors (CDDs) distributed albendazole door-to-door to all community members from the ages of 1­­–99 years excluding women in their first trimester of pregnancy, those with history of adverse reaction to benzimidazoles, and those acutely ill at the time of cMDA*”**

3) Explain the data collection tool based on each structure of the model and with what scale was it measured?

**The explanation about the data collection tools is included on page 7, line # 162-165 as “*Quantitative data were collected via a 37-item questionnaire consisting of 15 questions about background variables, 19 questions with 5-point Likert scale questions (‘strongly agree’ to ‘strongly disagree’) and three questions with 3-category nominal scale questions (‘yes’, ‘not sure’, and ‘no’)*”. The explanation about the scales used is included in page 8, line # 179-182 on page 8 as “*The responses to five-point Likert scale questions of ‘strongly agree’ to ‘strongly disagree’ were scored as 5 to 1, with 5 being the most positive response and 1 being the most negative response. The responses to the three category nominal scale questions of ‘yes’, ‘not sure’, and ‘no’, were scored as 5, 3, and 1, respectively*.”**

Reviewer #3

The article looks at Impact of adverse events during community-wide mass drug administration for STH, under the DeWorm3 trial site in southern India using the Theory of Planned Behaviour (TPB), to understand perceptions about participating in cMDA for STH and drivers of participation in subsequent STH cMDA programs after experiencing an adverse effect. The objectives are clearly outlined. They have used a multi-method approach was used, including quantitative and qualitative data collection, which is appropriate for this study. The study population is clearly defined. Sample size involved all with adverse effects. Analysis was appropriately done.
No issues on methods.

**Thank you very much for your kind feedback.**

**Reviewer #4**: (No Response)

RESULTS

Reviewer #1

1) The analysis presented clearly matches the analysis plan and the results are clearly presented. The figures are of sufficient quality and clarify though the repetition of certain charts is unclear to me.

**We have revised the text in figure 1 to give better clarity. We have ensured that figures and tables are not repeated.**

2) The findings of this study though not entirely novel are deeply insightful. Of significance is the influence of education, social status, and occupation on the intention to participate in future cMDA after experiencing AEs. It is noteworthy that in other MDA surveys, compliance among the educated tends to be lower than among the uneducated as demonstrated by this study.  The sub-topics in the results section, make the concept, hypothesis and results easy to understand and follow.

**Thank you very much for your kind feedback.**

3) The magnitude of the challenge of AEs during MDAs and particularly in albendazole treatment requires more information in the paper. Another phenomenon experienced during MDAs is the issue of side events that cannot be attributed to MDA but rather a co-incidental event often detected upon investigations. So the question of efforts made to isolate actual events of AEs from coincidental events needs to be clarified including the understanding the timing of the AEs post-treatment.

**In this study, we included all participants who reported an AE because fear of AE could lead to non-participation in future even if their AE was not related to MDA. We have discussed this in page 7, lines 152-154 as “*All 74 individuals who reported experiencing an AE were invited to complete a survey during March-April 2021, six months after completing the last cMDA*.”**

Reviewer #2:

Corrections related to the results section are written in the required corrections article

Comments from the manuscript:

1) The discussion should be according to the findings of the study. First, write the final result according to the general purpose and compare it with other similar studies in this field. For each of the constructs of the theory of planned behaviour, make a comparison with other studies according to the results obtained and state your reasons for these results.

**The key finding of this study are discussed in the first paragraph of the discussion section including references to other studies (page 22, line #449-457) “*In this study, more women than men and children reported experiencing AEs during MDA as also been observed in other countries as well as India. Participation in cMDA after an AE in this study was high, with only 12% refusing to participate in the next cMDA and only 4% refusing to participate in all subsequent cMDA rounds. These rates are similar to other studies that have evaluated the impact of AEs on participation in MDA programs; studies in Ethiopia and malaria in Gambia have reported refusal to participate in MDA ranging between 7­–33% as a result of individuals having experienced an AE*.”**

**Each of the constructs are discussed including references to other studies in three paragraphs on pages 23-24, line # 464-494)**

2) Write the strengths and weaknesses of this study. State the limitations of this study

**Strengths and limitation are included as a separate section (page 25, line # 517-529)**

Reviewer #3

1) results are clearly presented but Line 238 Fig 1. the figure is not clear presented; participated and refused are not clear shown; this can be improved. There are also some graphs and comments in the table (I think it was not finalized)

**We have revised figure 1 for better clarity. We have included final graphs and tables.**

2) Line 323: what about positive attitude association with family type (nuclear or extended)?

**The analysis showed no statistically significant association of positive attitude with the family type.**

Reviewer #4: (No Response)

CONCLUSIONS

Reviewer #1:

The conclusions are supported by the data presented with clearly stated limitations of the study. Discussions on how these data can be helpful to advance our understanding of the topic under study can however be made stronger with further justification on its utility to the global program. The study is definitely of significant public health relevance in informing the trajectory of the transition from control to elimination of soil transmitted helminths.

**Thank you for the suggestion. This study is of public health relevance as it looks at behavioural factors influencing intention to participate after an AE. The sentence is clarified in page 26, lines # 536-539 as “*In addition, research using the TPB framework with larger sample sizes would better inform about behavioural factors influencing intention to participate after an AE in STH MDA and other NTD programs, particularly those transitioning from control to elimination programs*.”**

Reviewer #2:

Corrections related to the conclusion section are written in the required corrections article.

1) The application of this study should be written for others

**The application of this study for MDA programs is included in page 26, line # 536-539 as “*Messages on AE and its management need to be incorporated in community mobilization activities and specifically aimed at castes other than SC/ST and nuclear families in MDA programs. CDDs involved in MDA programs need to be oriented about AEs during cMDAs as they are important stakeholders in mitigating the fear of AEs*”**

Reviewer #3:

This is clearly supported by the data presented. However, recall bias may have contributed to a lot of changes in these findings as 6 months after MDA is a long time to remember. Is it possible that there were no gender differences in attitudes due to recall bias?

**There could be recall bias particularly due the perceived severity of the AE. Our study did not show any gender difference in attitude, however similar studies with larger sample size may provide better evidence.**

Reviewer #4: (No Response)


EDITORIAL AND DATA PRESENTATION MODIFICATIONS

Reviewer #1:

Very useful conclusions and recommendations are made in the paper. Though these conclusions and recommendations are not entirely new they emphasize the utility of TPH in studying the three aspects of participants’ attitudes, subjective norms, and perceived behaviour control and an individual’s intention to participate in cMDA. This a Remarkable paper and should be published with or without the suggested modifications.

**Thank you very much for your kind feedback.**

Reviewer #2:

Corrections are written in the required corrections article

**All corrections have been addressed in the respective sections**

Reviewer #3:

Figure 1 need to be reworked

**Thank you very much for your feedback. We have revised figure 1 for better clarity**

Reviewer #4: (No Response)

SUMMARY AND GENERAL COMMENTS

Reviewer #1:

This manuscript should be of great interest to the global NTD community considering the inflection point of the global program with the current discussion and anticipated change from control to elimination. The impact of adverse events on control/elimination activities including mass drug administration is particularly critical. Furthermore, community-wide MDA as against school-based MDA has been recommended as the path forward towards the elimination of soil-transmitted helminthiasis. Very useful conclusions and recommendations are made in the paper. Though these conclusions and recommendations are not entirely new they emphasize the utility of TPH in studying the three aspects of participants’ attitudes, subjective norms, and perceived behaviour control and an individual’s intention to participate in cMDA. This a Remarkable paper and should be published with or without the suggested modifications.
Thank you very much for your kind feedback.

Reviewer #2:

Corrections are written in the required corrections article
**All corrections have been addressed in the respective sections**

Reviewer #3:

This was a good study, but it was mainly limited by the long period between the last MDA and the actual research. Some of the findings are likely to be affected by recall bias. Nevertheless there is still some good information obtained from this study.

- **Yes, the findings may be affected by recall bias as highlighted in our section on limitations of the study (page 25, lines # 526-528), “*there could be a recall bias as we carried out this research six months after completing all six rounds of cMDA due to delays driven by the COVID-19 pandemic*.”**

Reviewer #4:

Dear Authors, Here my comments:
1) Line 188-189: Please indicate here when Chi-square test or Fischer exact test were used or as footnote in the result table.

**All Fischer exact test are indicated in the table and referenced in the footnote.**

2) Line 190: Why call the four the TPB variables outcome variable here? Given the descriptive nature of chi-Square test, it is preferable to call them the dependent variables. As mentioned in Line 196-197 the intention variable is the outcome variable.

**Thank you for your feedback. We have revised the manuscript to include three TPB constructs - attitude, subjective norm, and perceived behavioural control as independent variables and intention to participate as the outcome variable (page 7, lines # 165-167) as “…*to measure the three independent variables (attitude, subjective norms, perceived behavioural control) and outcome variable (intention to participate)*”**

3) Line 190: Not necessary to quality path analysis as “multiple regression-based” …… To simply say path analysis with what it does is sufficient.

**Thank you. We have edited to keep it to path analysis (page 9, line # 199).**

4) Line 192: “This determines…. What is “This”? Please rephrase the sentence to make clearer.

**Thank you. We have clarified it as path analysis (page 7, line # 201).**

5) Line 199: How was G-power used to calculate the power of a statistical test? What is that “a” statistical test you used it for in your study? There is a need to have clear procedure here. What was used? Why and how?

**We used GPower 3.1 to calculate the power of a statistical test for the path analysis. The statistical power (0.97) was computed using an effect size (f^2^) of 0.35 (calculated to be large using Cohen’s f^2^) (Cohen J, 1988), at α = 0.05, with n = 59, and 3 predictors (page 9, lines # 208-210).**

6) I suggest you give a clear information here under data analysis section, how the minimum sample size required for the path analysis was estimated with references, rather giving a post-hoc power analysis as shown in your result section.  This is important given the small sample size (59) used in your path analysis.

**As the study population was limited to only 74 participants and due to the lack of literature on using TPB in the context of AEs in NTDs, we decided to include all in the study, and calculated a post-hoc power estimation.**

7) Line 202: “final codes arrived with consensus”, … Please check grammar.

**We have revised the sentence for better clarity on page 9, lines # 213-214 as ‘…. *the discrepancies in coding between the two primary coders were reviewed and revised with consensus…*.’**

8) Line 420-423. Please refer to my earlier comments on sample size. This information should be in data analysis section Please state the names of your predictors. And the reference/justification for the choice of your effect size of 0.35.

**Thank you for your suggestion. We have revised the analysis section incorporating your suggestions (page 9, lines # 208-210)** **as “*GPower 3.1 was used to calculate the power of a statistical test for the path analysis (33). The statistical power (0.97) was computed using an effect size (f^2^) of 0.35 (calculated to be large using Cohen’s f^2^), at α = 0.05, with n = 59, and three predictors.*”**

9) Strengths and limitations- There is a need to mention limitation regarding lack of generalizability of the study findings given the sample size and been part of a randomized trial with usually have a set of criteria that limits the inclusion of a section of the wider population.

**Thank you. We randomised clusters of villages, and as recommended by WHO and Government of India, except for children under the age of one year and women in their first trimester of pregnancy, all were included in the trial. The power of this study was high with a sample size of 59. However, results of this study cannot be generalized to all geographies and other NTDs. We have clarified in the lines page 25, #528-529 as follows “The findings of this study may not be generalisable to other geographies and other NTDs where similar studies would be required”**
