# Supplementary material for: Impact of adverse events during community-wide mass drug administration for soil-transmitted helminths on subsequent participation–a Theory of Planned Behaviour analysis
Source: PLoS Negl Trop Dis. 2023 Mar 14;17(3):e0011148. doi: 10.1371/journal.pntd.0011148 (PMC10013912; doi:10.1371/journal.pntd.0011148)
Supplement: Attachment — Submitted filename: Response to Reviewers.docx [file pntd.0011148.s005.docx]

February 6, 2023

Georgios Pappas and Eva Clark

Editors

PLOS Neglected Tropical Diseases (NTDs)

Re: PLOS Neglected Tropical Diseases submission (PNTD-D-22-01128)

Dear PLOS NTDs Editors,

We sincerely thank the reviewers for their comments on our manuscript titled: "Impact of adverse events during community-wide mass drug administration for soil-transmitted helminths on subsequent participation – a Theory of Planned Behaviour analysis." We have modified the manuscript in response to the comments and feedback. Our responses are given below.

Sincerely,

Sitara Swarna Rao Ajjampur

The Wellcome Trust Research Laboratory, Division of Gastrointestinal Sciences, Christian Medical College, Vellore, India

Phone: +91-416-228-2052

Fax: +91-416-228-2486

Email: [sitararao@cmcvellore.ac.in](mailto:sitararao@cmcvellore.ac.in)

RESPONSE TO REVIEWERS

METHODS
Reviewer #2: In the introduction section, the abbreviation can be written as a subtitle, but it must be according to the guidelines of the journal.

**Thank you for the suggestions. The manuscript we submitted includes all heading and subheading as per the journal guidelines.**

In the introduction section, the reason for using this theory should be explained more clearly in the introduction section of the study.

**To the description on page 5, line #100-111, we have included further clarification of TPB as, “*Change in any of these three predictors account for considerable variance in individual's intention to adopt a behaviour. TPB specifically includes perceived behavioural control as factors like money, time or other resources, and control by family member are barriers to individual's ability to access a service or adopt a behaviour. This theory provides a conceptual framework that can be applied to any behaviour of interest.”*** **(line # 107-111)**

The code of study ethics should be written. Mention how to comply with the ethical standards and the code of ethics in the work method.
**As specified by the journal guideline, this is included as a separate paragraph on pages 9-10, line# 219-225 as, *“The Institutional Review Board (IRB) of Christian Medical College (CMC), Vellore (10392 [INTERVEN]), and the Human Subjects Division at the University of Washington (STUDY00000180) approved the DeWorm3 trial in India. The trial is registered at ClinicalTrials.gov (NCT03014167). The CMC IRB approved this study on AE among Deworm3 trial participants as an amendment (IRB – A12, October 28, 2020). Written informed consent was obtained from all who participated in the survey and in-depth interviews. In case of children, the primary caregivers were interviewed with written informed consent.”***

RESULTS

Reviewer #2: (No Response)


CONCLUSIONS
Reviewer #2: Limitations of the study and strengths and weaknesses should also be corrected.
Many corrections requested by the referees have been modified by the authors.
**Yes, we revised to include all the corrections suggested by the reviewers.**

EDITORIAL AND DATA PRESENTATION MODIFICATIONS
Reviewer #2: (No Response)

SUMMARY AND GENERAL COMMENTS
Reviewer #2: (No Response)
